# Bending Rigidities and Interdomain Forces in Membranes with Coexisting Lipid Domains

**DOI:** 10.1016/j.bpj.2015.05.003

**Published:** 2015-06-16

**Authors:** Benjamin Kollmitzer, Peter Heftberger, Rudolf Podgornik, John F. Nagle, Georg Pabst

**Affiliations:** 1 University of Graz, Institute of Molecular Biosciences, Biophysics Division, NAWI Graz, Graz, Austria; 2 BioTechMed-Graz, Graz, Austria; 3 Department of Theoretical Physics, Jožef Stefan Institute, Ljubljana, Slovenia; 4 Department of Physics, Faculty of Mathematics and Physics, University of Ljubljana, Ljubljana, Slovenia; 5 Department of Physics, University of Massachusetts, Amherst, Massachusetts; 6 Department of Physics, Carnegie Mellon University, Pittsburgh, Pennsylvania

## Abstract

To precisely quantify the fundamental interactions between heterogeneous lipid membranes with coexisting liquid-ordered (Lo) and liquid-disordered (Ld) domains, we performed detailed osmotic stress small-angle x-ray scattering experiments by exploiting the domain alignment in raft-mimicking lipid multibilayers. Performing a Monte Carlo-based analysis allowed us to determine with high reliability the magnitude and functional dependence of interdomain forces concurrently with the bending elasticity moduli. In contrast to previous methodologies, this approach enabled us to consider the entropic undulation repulsions on a fundamental level, without having to take recourse to crudely justified mean-field-like additivity assumptions. Our detailed Hamaker-coefficient calculations indicated only small differences in the van der Waals attractions of coexisting Lo and Ld phases. In contrast, the repulsive hydration and undulation interactions differed significantly, with the latter dominating the overall repulsions in the Ld phase. Thus, alignment of like domains in multibilayers appears to originate from both, hydration and undulation repulsions.

## Introduction

Diverse physiological processes in living systems depend on fundamental physical interactions between lipid membranes acting on the nanoscopic length scale. Of particular interest in this context are, in addition to intramembrane interactions (1,2), forces acting between membrane domains/rafts across the aqueous phase, which are also involved in their correlated mutual alignment. Such positional correlations are well established for liquid-ordered (Lo)/liquid-disordered (Ld) domains in model lipid multibilayers (3–11). Several groups have established compositional phase diagrams for mixtures of high-melting lipid, low-melting lipid, and cholesterol, which exhibit Lo/Ld phase coexistence over a broad range of compositions and temperatures (12,13). These systems mimic mammalian outer plasma membranes and make it possible to study domain properties under well-defined conditions. Most recently, we reported structural details of Lo/Ld phases in two ternary lipid mixtures using a global small-angle x-ray scattering (SAXS) analysis for coexisting lipid domains (11). This analysis relies on the above-mentioned mutual alignment of like domains. However, domain alignment is also of biological relevance, for example, in the context of the immune response, where organization of receptor-ligand domains occurs during T-cell adhesion (14,15). Both the formation of such domains and the adhesion affinity depend strongly on thermal fluctuations and, consequently, on the bending rigidity of membranes (16,17). It is therefore reasonable to expect that fundamental intermembrane interactions will play an important role also in receptor-ligand domain alignment.

Within the broad Derjaguin-Landau-Verwey-Overbeek (DLVO) paradigm (18), the fundamental long-range interactions between soft material interfaces, mediated by their molecular environment, such as solvation (hydration) interaction, electrostatic interaction, and van der Waals interaction, can be treated independently and additively. However, this additivity Ansatz is in general not vindicated for entropically driven bending undulation interactions, which warrant a more sophisticated approach (18–20).

Besides the fundamental role of entropic membrane undulations, their relation with the membrane bending rigidity, *K*_c_ (19), and through it their connection with diverse physiological processes, has spurred a sustained scientific interest (21). Shape analysis of giant unilamellar vesicles (GUVs) (22), diffuse x-ray scattering from oriented lipid multibilayers (23), and GUV micropipette aspiration (24) are all techniques exploiting this connection, but so far, none of them has been able to simultaneously determine the bending moduli for coexisting membrane phases. On the other hand, macroscopically sized domains form distinct lamellar lattices in multibilayer systems, making it possible to apply osmotic stress experiments (8,25). In such experiments, osmotic pressure is maintained by, e.g., large neutral polymers, such as polyethylene glycol (PEG), which do not penetrate into the interbilayer water layer, whereas the corresponding bilayer separation and more recently also the specific line broadening due to fluctuations are measured by SAXS. Several groups, including ours, have previously applied this approach to study interactions between macromolecules, including lipid bilayers (8,25–34).

The bare long-range DLVO interaction components, which couple macromolecular surfaces through their molecular environment, get inextricably intertwined through the thermally driven conformational fluctuations of the soft interfaces, making detailed predictions of the overall interaction nearly impossible. Therefore, many studies in the past have resorted to describing such complicated thermal fluctuation effects by different mean-field/additivity approximations, where conformational fluctuation effects on the bare interaction potentials are included self-consistently (19,20,35–37). In contrast, additivity/mean-field approximations can be altogether avoided in the case of simulations that start from fundamental long-range DLVO interaction components and need no additional approximations to yield an accurate estimate for the total osmotic pressure in the system (38,39).

To understand the coupling between bare interactions and thermal undulations in phase-separated systems, we apply a gradient-based optimization algorithm to iteratively adjust the parameters in Monte Carlo (MC) simulations, i.e., the coefficients describing the strength and range of intermembrane interactions, as well as the bending rigidity characterizing the thermal undulations, to best match simulation results with the experimental osmotic stress data for coexisting Lo/Ld phases. We demonstrate the capability of the simulation-driven analysis choosing a well-studied mixture of dioleoyl phosphatidylcholine (DOPC), distearoyl phosphatidylcholine (DSPC), and cholesterol (Chol) (40–42), previously shown to exhibit Lo/Ld domain alignment in the phase-coexistence regime (11). We find that Lo domains are about three times more rigid than Ld domains, which exhibit significant contributions to domain repulsion from bending fluctuations. On the other hand, hydration forces decay much slower with domain separation between Lo domains. In turn, attractive van der Waals interactions were found to be of similar magnitude between Lo domains and between Ld domains. Our results provide insight into the strength and distance dependence of forces at play between like domains as a prerequisite to devising theories for domain alignment.

## Materials and Methods

### Sample preparation

DSPC, DOPC, and Chol were purchased from Avanti Polar Lipids (Alabaster, AL) and used without further purification. PEG with an average molecular weight of 8000 was obtained from Fluka Chemie (Buchs, Switzerland) and used as received.

After weighing, lipids were dissolved in chloroform/methanol 2:1 at concentrations of 10 mg mL^-1^. The supplier-provided molecular weights, accounting for an additional water molecule with DOPC, were used for determining stock concentrations. We prepared the ternary lipid-only mixture DOPC/DSPC/Chol (0.42:0.37:0.21) in a glass vial and evaporated the organic solvent under a gentle nitrogen stream at 30°C. This lipid composition and its tie line lie well inside the Lo/Ld phase-coexistence region according to Zhao et al. (40) and Heberle et al. (41), and the domains’ structural properties have already been investigated by different methods (11,42). The remaining solvent traces were removed by placing the samples in a vacuum overnight. The next day, 18 MΩ cm water (UHQ PS, USF Elga, Wycombe, United Kingdom) was added at 20 *μ*L water/mg lipid and the mixtures were fully hydrated at 50°C for 4 h with repeated freeze-thaw cycles.

To exert osmotic pressure on multilamellar lipid vesicles, samples were cooled to room temperature after hydration and aliquots were overlaid with PEG dissolved in water, yielding final concentrations of 1–42 wt% PEG in water. Samples were protected against oxidation with argon, and the vials were closed, taped, and stored at 4°C for 7–10 days prior to measurement. The osmotic equation of state for PEG, connecting its osmotic pressure with its solution concentration, is well known (43) and allows for an accurate determination of PEG osmotic pressure *P* by using previously reported high-resolution data (44).

### X-ray measurements

SAXS was performed at the Austrian SAXS beamline at ELETTRA, Trieste, Italy (45,46), at a wavelength of 1.54 Å and an energy dispersion, Δ*E*/*E*, of 2.5 × 10^−3^. We used a mar300 Image Plate 2D detector (marresearch, Norderstedt, Germany) covering a *q*-range from 0.2 to 7.1 Å^−1^ and calibrated with silver-behenate (CH_3_(CH_2_)_20_−COOAg) with a *d*-spacing of 5.838 nm (47). Samples were filled into reusable quartz-glass capillaries and kept in a brass sample holder connected to a circulating water bath (Huber, Offenburg, Germany). The samples were equilibrated for 10 min at (20.0 ± 0.1)°C before exposing them for 30 s to the x-ray beam.

The two-dimensional detector signal was radially integrated with FIT2D (48,49). Water background subtraction for samples without PEG was performed with Primus (50). For osmotically stressed samples however, additional scattering from PEG made a standard background subtraction impractical. Since the essential information in this case was the shapes and positions of the Bragg peaks, we subtracted approximate backgrounds, obtained by interpolating between SAXS signals of pure water and PEG/water mixtures. Alternatively, one could just subtract an arbitrary smooth function from the measured spectra.

The reduced data were then fitted using a recently published, full *q*-range analysis method for coexisting liquid/liquid membrane domains (11). We checked the x-ray analysis for coexisting phases by comparing it with PEG-free, homogeneous samples prepared at the published tie-line endpoint concentrations of 0.79:0.09:0.12 for the Ld and 0.05:0.65:0.30 for the Lo phase (41). These samples were also helpful for constraining some model details (e.g., the widths and distances between molecular subgroups composing the lipid heads) in the x-ray analysis.

For the x-ray analysis, the contribution of each individual phase is modeled with a bilayer structure and a superimposed membrane lattice. The lattice description is based on a modified Caillé theory (51,52) and therefore yields the average membrane periodicity, *d*, and the line shape parameter, *η*, which is connected to the mean-square fluctuation of the membrane spacing via Δ^2^ = *ηd*^2^/*π*^2^ (32). The bilayer structure of each phase is then modeled separately via probability distributions of quasi-molecular fragments (53).

Of most importance, the full *q*-range analysis allowed us to quantify the magnitude of fluctuations for coexisting domains. For both phases of stress-free samples, this also yields accurate electron density profiles, from which the bilayer thickness could be obtained; but this was not possible when osmotic pressure was applied. Instead, the osmotic thickening of *d*_B_ was calculated using *d*_B_(*P*) = *d*_B_(0) × (*K*_A_ + *P* × *d*(*P*))/(*K*_A_ + *P* × *d*_B_(0)) (31), where the area extension modulus, *K*_A_, was estimated from published micropipette aspiration experiments on single lipids and binary lipid mixtures (54,55), as detailed in Section S1 of the Supporting Material. The overall analysis was rather insensitive to uncertainties in *K*_A_, because the maximal change in bilayer thickness was only slightly larger than the uncertainty of the fit (±2%). The definition of the bilayer thickness, *d*_B_, was found to be more important. In principle, one could determine optimal values of *d*_B_ via a joint fit with free MC parameters, but this problem is underdetermined and led to bizarre values of *d*_B_ for different data sets (56). Instead, we defined *d*_B_ as the distance between the remotest lipid atoms, also known as the steric bilayer thickness (29); this yielded good fits and comparable results and at the same time was directly accessible from the SAXS analysis. Specifically, we used *d*_B_ = 2(*z*_CholCH3_ + *σ*_CholCH3_), where *z*_CholCH3_ and *σ*_CholCH3_ are the position (measured from the bilayer center) and the width, respectively, of the CH_3_ groups in the lipid head choline. Within measurement accuracy, the definition used in Petrache et al. (32) yields equal values.

### Membrane MC simulation

The simulation code used has been described previously in detail for a single membrane between two walls and for a stack of membranes (38,39,56). For completeness, but also to highlight our modifications, we briefly summarize its basic elements.

**Figure 1:**
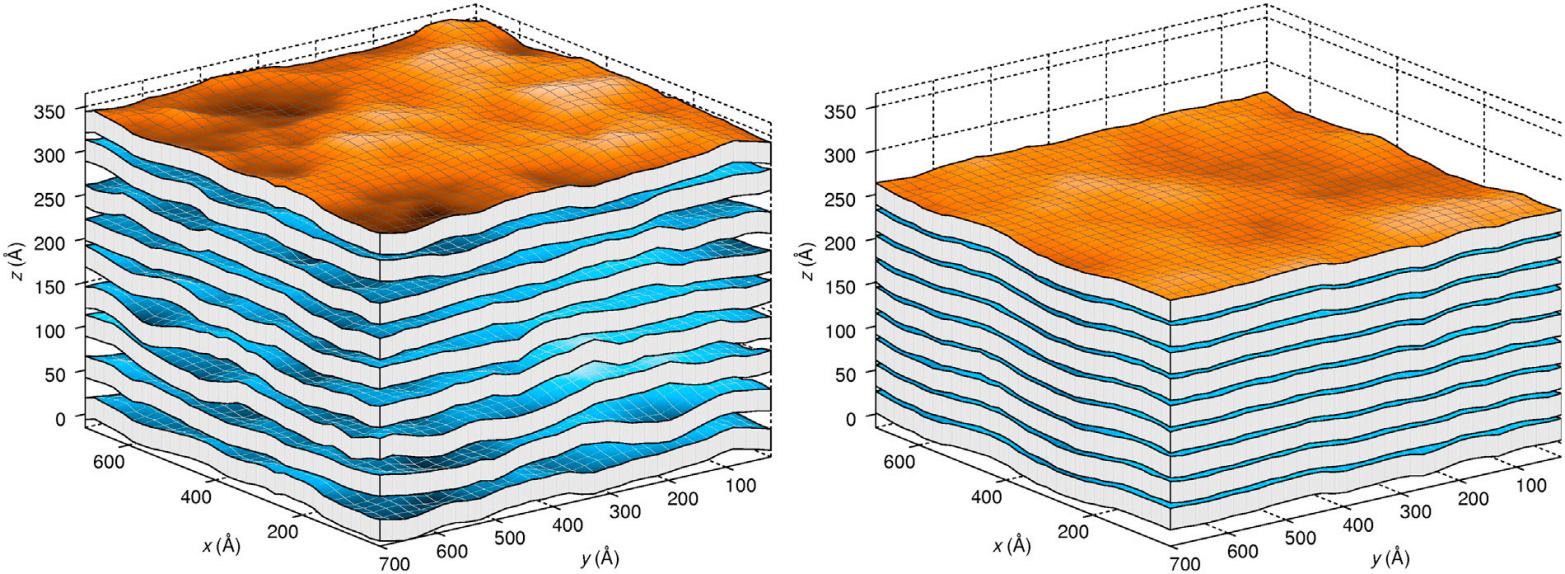
Real-space snapshots of equilibrated Ld simulations at zero (*left*) and finite (5.5 MPa) osmotic pressure (*right*). Membranes are drawn with their average thickness. Deviations from the periodic lattice are color coded. Due to 3D periodic boundary conditions, the top-most (*orange*) and bottom-most membranes are equal. The most prominent effects of external pressure, a compression of the stack and a reduction of the fluctuations, are clearly visible. To see this figure in color, go online.

The system under consideration consists of a stack of *M* fluctuating and interacting membranes of size *L* × *L*, as depicted in Fig. 1. The displacement of the *m*th membrane from its average plane is denoted as *u*_*m*_(*x*,*y*), the average distance between membranes as $\bar{a}$, and the bending rigidity as *K*_c_. Imposing periodic boundary conditions in all directions yields the Hamiltonian of a stack of membranes:

(1) $$H=\sum_{m=0}^{M-1}\int \left(\frac{K_{c}}{2}{\left(\nabla^{2}u_{m}\right)}^{2}+\text{Φ}\left(a_{m}\right)\right)dx\;dy,$$

where Φ denotes the bare interaction potential, given here by the hydration repulsion and the van der Waals attraction, and $a_{m}\left(x,y\right)=u_{m+1}\left(x,y\right)-u_{m}\left(x,y\right)+\bar{a}$ denotes the local distance between two membranes. We furthermore require that *a*_*m*_ ≥ 0, meaning that membranes cannot interpenetrate.

To reduce the degrees of freedom of the system to a finite amount, the membranes are discretized on a square *N* × *N* lattice. The simulation is performed in the constant pressure ensemble (57), which converges for this model faster than constant volume simulations (39). MC updates are proposed in $\bar{a}$ and in the complex coefficients *u*_*m*_(*q*_*x*_,*q*_*y*_) of the Fourier transformation of *u*_*m*_(*x*,*y*). Simulating in Fourier space allows for larger moves, thereby accelerating equilibration (39). After every MC step (MCS), which corresponds to degree-of-freedom (*N*^2^*M* + 1) update proposals, we recentered the coordinate system to correct for small center-of-mass movement as a new feature in the calculations.

Simulations were performed for *L* = 700 Å, several different *N* in {6,8,12,16,24,32}, *M* = 8, equilibration lengths of 3×10^3^ MCS, and collection lengths of 10^4^ MCS, which typically exceeded the autocorrelation time by a factor of 100. Simulations were started with MCS sizes estimated from an approximative theory (20) and then subsequently optimized during equilibration, applying either dynamically optimized MC (DOMC), or—as a new feature—the acceptance ratio method (ARM) as a backup if DOMC fails (56,58).

Several observables can be determined from converged simulations, but the two most important quantities for comparison with SAXS experiments are the temporally and spatially averaged distance between membranes $d_{W}=\langle \bar{a}\rangle$ and the time average of its fluctuations,

(2) $$\Delta^{2}=\langle \bar{{\left(z_{m+1}\left(x,y\right)-z_{m}\left(x,y\right)-d_{\text{B}}-d_{\text{W}}\right)}^{2}}\rangle ,$$

where the long bar denotes spatial averaging over (*m*,*x*,*y*), angled brackets denote time averaging, and $z_{m}\left(x,y\right)=u_{m}\left(x,y\right)+m\times \left(\bar{a}+d_{\text{B}}\right)$ is the position of the *m*th membrane in real space. Specifically, *d*_W_ corresponds to the experimental thickness of the water layers separating the lipid bilayers, whereas Δ is related to the experimental Caillé parameter *η*, as discussed above.

The computed observables change significantly with *N*/*L* (38,39), so simulations were performed for a sequence of values of *N* and the observables were then extrapolated toward *N*/*L* → ∞. Further details of this finite size convergence are given in Section S2 in the Supporting Material.

It should be emphasized that our explicit purpose of making contact with the x-ray structure factor and the interactions between bilayers requires much larger systems than can be presently envisioned either for all-atom simulations, used to obtain electron density profiles, or even for the most coarse-grained molecular simulations (59). We require *M* bilayers in a stack, each bilayer having a large lateral size *L*. It has been shown in previous work (38) that *L* = 700 Å and *M* = 8 are sufficient to obtain accuracies of 1% for *d*_W_ and Δ, and that would require ∼130,000 lipids with associated water in typical molecular simulations. Apart from simulation size, the necessary timescales, which scale with the fourth power of the undulation wavelength (see pp. 77–78 in Pabst et al. (60)), render molecular dynamics simulations for that purpose unfeasible. Furthermore, to fit the experimental data requires on the order of 100 separate simulations, distributed on multiple optimizations from different start points. In the membrane MC simulations we employ, each bilayer is reduced to a network consisting of *N* nodes in each of the two lateral directions, and each node has only one degree of freedom.

### Bare interaction potentials

For uncharged membranes, the potential at bilayer separation *a* is modeled canonically by

(3) $$\text{Φ}\left(a\right)≃A\lambda \text{exp}\left(-\frac{a}{\lambda }\right)-\frac{H}{12\pi a^{2}}.$$

The first term in Eq. 3 is the well-established empirical form of the solvent-mediated hydration interaction, which has been argued to originate from changes in various measures of order for the water structure at the membrane interface (62–64), with interaction strength *A* and decay length *λ*, which is typically in the range of 1–2 Å (32). The second term describes the ubiquitous van der Waals interaction potential for two planar semiinfinite layers, with *H* being the Hamaker coefficient, which in general also depends on bilayer separation *a*, *H* = *H*(*a*) (see p. 15 in Parsegian (65)). This functional form is convenient because it can in fact describe the cases of either two finite-thickness layers interacting across a solvent layer or effective pairwise interactions in an infinite stack of finite-thickness layers (66). For large solvent layer thickness, the nonpairwise additive effects in the latter case become negligible and the van der Waals interaction potential for the two cases follows exactly the same separation dependence.

Due to the divergence of the van der Waals potential for *a* → 0, the 1/*a*^2^ term is cut off for *a* < 1 Å (38). In experiments, the collapse of charge-neutral bilayers due to van der Waals forces is avoided by very short-range steric interactions established by McIntosh et al. (61) that occur at significantly higher osmotic pressures than those relevant for the experiments presented here (see also Fig. S5). Although we added such an additional steric repulsion of the form *A*_st_*λ*_st_ exp( − *a*/*λ*_st_) to Eq. 3, with *A*_st_ = 3.6 GPa and *λ*_st_ = 0.6 Å according to (61), it proved unimportant for realistic parameters.

**Figure 2:**
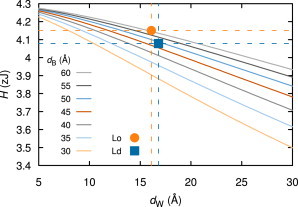
Hamaker coefficient, *H*, for hydrocarbon multilayers of height *d*_B_ and separation *d*_W_ in water. Highlighted are the applied values of *H* for Ld and Lo, which are described in the main text. To see this figure in color, go online.

To calculate the Hamaker coefficient, *H*, ab initio, we had to approximate the lipid bilayers by pure hydrocarbon. Although this model gives only a first-order estimate for the van der Waals interactions of fluctuating lipid bilayers, it is, to our knowledge, the best available approximation in the absence of data on the dielectric response of PC lipids. Further effects of, e.g., lipid-headgroup dipolar-moment fluctuations (67), could be considered as well, but they would be important only at very small separations where hydration forces dominate and the exact form of the van der Waals interaction is irrelevant. Specifically, we calculated *H* for an infinite stack of hydrocarbon layers in water, based on a full multilayer Lifshitz formulation (66). The ranges for the hydrocarbon thicknesses, *d*_B_ = 45–60 Å, and the water spacings, *d*_W_ = 5–60 Å, were motivated by our experimental data. In this calculation range, differences in the Hamaker coefficient were within 10%. For our MC simulations, the exact value of *H* matters most when all forces are of comparable magnitude, that is, at vanishing external osmotic pressure. We therefore used the *H* values of 4.08 × 10^−21^ J = 4.08 zJ for Ld and 4.15 zJ for Lo domains (see Fig. 2).

Both components of the bare potential, i.e., hydration and van der Waals interactions, cause partial bare pressures between neighboring membranes given by

(4) $$P_{\text{hyd}}\left(d_{\text{W}}\right)=A\text{exp}\left(-\frac{d_{\text{W}}}{\lambda }\right),P_{\text{vdW}}\left(d_{\text{W}}\right)=-\frac{H}{6\pi d_{\text{W}}^{3}}.$$

Equation 4 was derived from $P_{\text{j}}\left(d_{\text{W}}\right)\approx -\partial {\text{Φ}}_{\text{j}}\left(d_{\text{W}}\right)/\partial d_{\text{W}}$. The difference from the exact relationship, $P_{j}\left(d_{W}\right)=\langle -\partial \text{Φ}\left(\bar{a}\right)/\partial \bar{a}\rangle$, was found to be less than the simulational uncertainty. For comparison to previous reports using mean-field/additivity approximations for modeling undulation interactions, one can obtain an effective decay constant *λ*_und_ by subtracting bare contributions from experimental data, i.e., *P*_und_ = *P* − *P*_hyd_ − *P*_vdW_ (39). The undulation decay constant then results from a fit of *P*_und_ = *A*_und_ exp( − *d*_W_/*λ*_und_), with the two adjustable parameters *A*_und_ and *λ*_und_. Because the undulation pressure deviated significantly from a perfect exponential, we limited the fit to large separations (*d*_W_ ≥ 14 Å).

### Optimizing parameters against experimental data

Calculation of the Hamaker coefficient *H*, as described above, allowed us to reduce the number of free-fitting parameters for the simulations to three, $\vec{\Lambda }=\left(A,\lambda ,K_{\text{c}}\right)$, for a joint analysis of domain separation and fluctuation data (see below).

We implemented a least-squares routine with Matlab (68), utilizing its trust-region reflective optimization algorithm to minimize the sum of the squared residues

(5) $$\chi^{2}\left(\vec{\Lambda }\right)=\sum_{i}{\left(\frac{d_{\text{W},i}-d_{\text{W}}\left(P_{i};\vec{\Lambda }\right)}{U_{\text{eff}}\left(d_{\text{W},i}\right)}\right)}^{2}+{\left(\frac{\Delta_{i}-\Delta \left(P_{i};\vec{\Lambda }\right)}{U_{\text{eff}}\left(\Delta_{i}\right)}\right)}^{2},$$

where *d*_W,*i*_ and Δ_*i*_ are the experimentally determined values at fixed osmotic pressure *P*_*i*_, $d_{\text{W}}\left(P_{i};\vec{\Lambda }\right)$ and $\Delta \left(P_{i};\vec{\Lambda }\right)$ are simulation results, and *U*_eff_(*f*) is the effective uncertainty of a given quantity *f*, derived from

(6) $$U_{\text{eff}}^{2}\left(f\right)=U^{2}\left(f_{\text{exp}}\right)+U^{2}\left(f_{\text{sim}}\right)+{\left(\frac{\partial f_{\text{sim}}}{\partial P}\times U\left(P_{i}\right)\right)}^{2}.$$

The agreement between model and data was evaluated by the reduced *χ*^2^ value, $\chi_{\text{red}}^{2}=\chi^{2}/\tilde{N}$, where $\tilde{N}$ equals the number of data points minus the number of free parameters (see p. 268 of Taylor (69)). The Jacobian for this gradient-based algorithm and the derivative in Eq. 6 were computed with the histogram reweighting method described in Section S3 in the Supporting Material. Once the iteration converged, the uncertainties of the fit parameters were determined from the curvature of $\chi_{\text{red}}^{2}$. To locate the global optimum, several iterations from randomly chosen initial parameter sets were performed.

To test our implementation, we fitted simulation results determined for one reasonable parameter set, ${\vec{\Lambda }}^{\prime }$, by starting the least squares from several different initial starting points $\vec{\Lambda }$. Within three to five iterations, these optimizations converged toward the correct values, ${\vec{\Lambda }}^{\prime }$, thereby indicating that the weighted-histogram-based differentiation and the fit were correctly implemented. For the experimental data sets, convergence was usually reached within 10 iterations. However, due to the stochastic nature of the simulations and the consequential randomness of results and derivatives, the optimization algorithm propagated poorly in flat regions, i.e., small $\vec{\nabla }\chi_{\text{red}}^{2}$. Because $\chi_{\text{red}}^{2}\left(\vec{\Lambda }\right)$ is a smooth function and its gradient has to vanish at extrema, the efficiency of the optimization algorithm decreased the closer it got to the optimum. This was another reason for starting several independent iterations. Alternatively, one could have used optimization algorithms specialized for simulations (70–73), but the existing implementations did not satisfy our needs.

As a further test case, we reanalyzed previously published osmotic pressure data of pure dimyristoyl phosphocholine (DMPC) bilayers (32), yielding very reasonable values and a good agreement between simulations and experiments. Details are given in Section S4 in the Supporting Material. Thus, we conclude that our method provides a robust analysis for interactions in fluctuating membrane assemblies.

## Results and Discussion

### X-ray analysis

**Figure 3:**
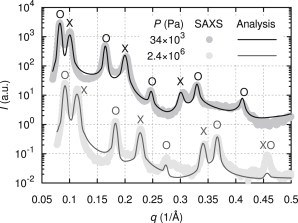
Calculated scattering intensities (*solid lines*) from full *q*-range analyses, compared with recorded SAXS data from coexisting phases (*dots*) for two different osmotic pressures, *P*. Bragg reflections from aligned Lo and Ld domains are indicated by symbols O and X, respectively.

SAXS patterns were analyzed as detailed previously by a Caillé theory-based analysis (11). Fig. 3 showcases the analysis for two samples at osmotic pressures of 34 kPa and 2.4 MPa, demonstrating that shapes and positions of Bragg reflections are well reproduced. Consistent with previous studies (8,9,11), we find sharper and more prominent Bragg reflections for the Lo phase due to its decreased bending fluctuations, compared to the coexisting Ld phase. Fits for all other samples are shown in Section S5 in the Supporting Material. For increased osmotic pressures, Bragg peaks shifted toward higher *q* and became more prominent. This is due to the decrease of bilayer separation associated with a reduction of bending fluctuations, in agreement with previous reports (32,74).

Peak line shapes for Lo and Ld domains were found to be well described by the applied Caillé theory, particular at low osmotic pressure (Fig. S4). Since this theory is incapable of fitting peaks from lamellar gel phases (75), we conclude that neither peaks assigned to the Lo nor those assigned to the Ld phase can originate from a gel phase. This is also consistent with compositional DSPC/DOPC/Chol phase diagrams reported in the literature (40,41) and a recent SAXS study from our lab, which reported for the identical lipid mixture that the structural parameters match those of Lo and Ld phases at the tie-line endpoints (11).

Fit quality of SAXS spectra worsened for increased PEG concentrations (see Fig. 3 or Section S5 in the Supporting Material). The underlying Caillé theory probably loses its applicability for the increased order experienced at elevated osmotic pressures. Although the effects on domain separation were negligible, fluctuations determined from the fits became increasingly skewed with osmotic pressure, in particular for Lo domains (see below).

**Figure 4:**
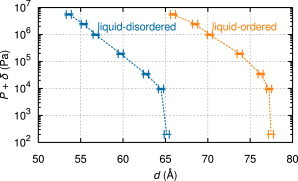
Osmotic pressure, *P*, versus membrane periodicity, *d*, for Ld and Lo determined by SAXS analysis. Dashed lines are meant solely as a guide for the eye. The small offset, *δ* = 200 Pa, is necessary for plotting *P* = 0 on a logarithmic scale. To see this figure in color, go online.

The effect of osmotic pressure on the lamellar repeat spacing, *d*, as determined from the SAXS analysis, is plotted in Fig. 4. At high osmotic stress, the distance between bilayers is effectively set by the repulsive hydration interaction, which dominates the repulsive fluctuation interaction and the attractive van der Waals interaction. As osmotic pressure is decreased, the water spacing between bilayers, *d*_W_, increases and the fluctuation interaction eventually dominates the hydration interaction. As the osmotic pressure is reduced to zero, the attractive van der Waals force balances the total repulsive forces, resulting in finite *d*_W_ and *d* values.

Within experimental uncertainty, the two isotherms in Fig. 4 are rather similar when the difference in membrane thickness, determined by $d_{\text{B}}^{\text{Ld}}=\left(48.5\pm 1.0\right)Å$ and $d_{\text{B}}^{\text{Lo}}=\left(61.3\pm 1.2\right)Å$, is considered. Of course, identical isotherms would imply that all the interactions are identical. However, significant experimental differences were observed in the fluctuation behavior, as detailed below, corroborating the crucial advantage of jointly analyzing fluctuations and osmotic pressure isotherms to obtain the interaction parameters (32).

### Optimized simulations

**Figure 5:**
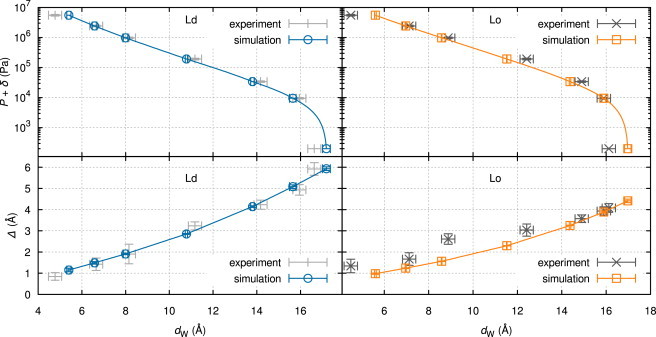
Osmotic pressure (*top*) and fluctuations (*bottom*) vs water-layer thickness for best fit of membrane MC simulation (*cyan*/*orange*) against SAXS data (*gray*). Solid lines were obtained by exponentially interpolating fluctuation contributions. The small offset, *δ* = 200 Pa, is necessary for plotting *P* = 0 on a logarithmic scale. To see this figure in color, go online.

The experimental data and the results of optimized simulations are compared in Fig. 5, and Table 1 lists results for the interaction parameters. Experimental errors for *d*_W_ and *η* were obtained from the SAXS analysis and those for *P* were estimated to equal the pipetting error of 6% for viscous PEG solutions. To quantify the agreement between data and simulations, we report $\chi_{\text{red}}^{2}$, which becomes ∼1 if the differences are compatible with experimental errors (see p. 268 in Taylor (69)). This is the case for the Ld phase, where simulations and experimental data match ideally, but the mismatch for Lo is larger than expected ($\chi_{\text{red}}^{2}=6$).

We are inclined to attribute this discrepancy for Lo at least partially to the limited applicability of the Caillé theory for highly ordered systems, as described in the previous section. Indeed, deviations in Δ are especially pronounced for small bilayer separations, i.e., at high osmotic pressures. In light of these discrepancies, we suggest that the experimental uncertainties determined for the Lo phase are rather too small, because they do not take into account the decreasing applicability of the Caillé theory for more ordered phases whose fluctuations are suppressed by low hydration.

Although differences in *P*(*d*_W_) are insignificant between Ld and Lo (see also Fig. 4), fluctuations of the Lo phase, containing most of the DSPC and about thrice as much cholesterol as Ld, are evidently smaller (Fig. 5). In the continuum mechanics treatment used in the simulations, this increase in bilayer stiffness is captured by a threefold-higher *K*_c_ for Lo (see Table 1).

The values obtained by us for *K*_c_ compare well with previously reported results obtained by different techniques. Several groups have measured the bending rigidities of binary DOPC/cholesterol mixtures, which ranged from 60 ± 8 to 100 ± 25 zJ and were found to be largely unchanged by the cholesterol content (76–79). This supports the *K*_c_ = (44 ± 10)zJ obtained for Ld, where DOPC is the main constitutent (41). In contrast, a larger concentration of saturated lipids, for which *K*_c_ does increase with cholesterol (76), is present in the Lo phase, so a larger bending rigidity would be expected for Lo than for Ld. Our finding of *K*_c_ = 120 ± 20 zJ for the Lo phase is consistent with this expectation.

Furthermore, molecular dynamics simulation results are available for comparison. Khelashvili et al. (80) used the reported tie-line endpoint compositions (41) to separately simulate the Ld and Lo phases, obtaining bending moduli of 80–130 zJ for Ld and 340–440 zJ for Lo. Although these values are large compared to our results, both methods find a strong increase of *K*_c_ between Ld and Lo.

In agreement with Pan et al. (81), we find that a rather simple model suffices to relate bending to area extension moduli for cholesterol-rich samples (82). Based on the assumption that the main contribution to membrane rigidity comes from the stiff cholesterol ring of size $\delta^{\prime }$, Pan et al. used the relationship $\delta^{\prime 2}=12K_{\text{c}}/K_{\text{A}}$. For our samples, with *K*_A_ = 430 mN m^-1^ and 2100 mN m^-1^ (see Section S1 in the Supporting Material for details), this equation yields $\delta^{\prime }$ = 11 Å and 8 Å for Ld and Lo, respectively, in good agreement with actual cholesterol ring sizes of ∼9 Å, giving additional support to our analysis.

### Interdomain forces

**Figure 6:**
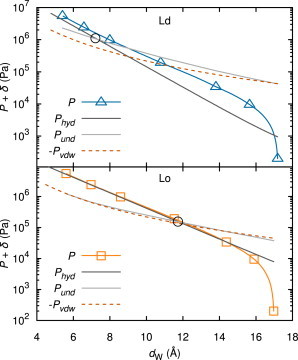
Partitioning of total pressure, *P*, into contributions from hydration, *P*_hyd_, van der Waals, *P*_vdw_, and undulation interactions, *P*_und_, for Ld (*upper*) and Lo (*lower*). The large open black circles show the values of the separation, *d*_W_, at which hydration and undulation pressure are equal. The small offset, *δ* = 200 Pa, is necessary for plotting *P* = 0 on a logarithmic scale. *δ* is also responsible for the deviation of the hydration pressure from a straight line at low *P*. To see this figure in color, go online.

As stated previously, the differences between Ld and Lo in the *P* versus *d*_W_ data sets are small. However, a more thorough investigation of these quantities yields interesting insights. Because good fits to these data were obtained, the total pressure *P* is readily dissected into its individual contributions from the fundamental surface forces, whose functional dependences are plotted in Fig. 6.

The thicker Lo bilayer causes an increase in the Hamaker coefficient, but only by 3% compared to that of the Ld phase; this is a minor difference in the van der Waals interaction that is hardly noticeable in the *P*_vdW_ curve in Fig. 6. For small bilayer separations, the hydration interactions are of similar magnitude and represent, as expected, the dominant contribution to the total interaction potential for both phases. Despite these similarities, the fluctuation pressure starts to surpass the hydration pressure already at much smaller separations, *d*_W_, for Ld than for Lo. This difference implies that, in contrast to the ordered phase, the undulation interaction becomes the most important repulsive interaction over a wider range of bilayer separations in the case of the disordered phase. Stronger repulsions due to fluctuation interactions are of course reasonable because thermal undulations were found to be significantly increased for the Ld phase (Fig. 5). Nevertheless, even in the Lo phase, the thermal undulation interaction dominates the hydration force over the most important, well hydrated range of *d*_W_, starting at separations of 12 Å.

We obtained almost exponentially decreasing fluctuation forces of the scaling form ∝ exp( − *z*/*λ*_und_), with effective decay lengths of *λ*_und_ ≈ 3.3 Å and 3.7 Å for Ld and Lo, respectively. The ratio of fluctuation to hydration decay length *λ*_und_/*λ* is obtained as 2.4 for Ld and 2.1 for Lo. Mean-field theory predicted this ratio's value as 2.0 (20), but values of 2.4 and 2.0–3.0 have been reported from simulations (38,39) and from other experiments (8,32,33), respectively.

Compared to Lo, a significantly shorter decay length for the hydration interaction pressure was found for the Ld phase. At present, the origin for this difference is unclear. However, it is this difference combined with the larger fluctuation force that gives *P* versus *d*_W_ curves that are nearly the same for Lo and Ld, both with fully hydrated *d*_W_ close to 17 Å.

Domain alignment across interlamellar aqueous phases has recently been hypothesized to be caused by water-network mismatch due to the different hydration properties of Lo and Ld phases (3). In support of this postulation, we observed significantly different hydration forces and nearly equal van der Waals forces for both phases. The aforementioned hypothesis neglected, however, influences from thermal undulation interactions, which we now find to differ considerably between coexisting Lo and Ld domains. The importance of thermal fluctuations is especially striking near full hydration, where undulation and van der Waals pressures surpass hydration repulsion by an order of magnitude (see Fig. 6).

## Conclusions

We have evaluated the fundamental long-range interactions between coexisting Lo and Ld domains in DOPC/DSPC/cholesterol, which is a frequently used model system for mammalian outer plasma membranes (11–13,40–42,82,83). Because we could do this at concentrations where Lo and Ld domains coexist, we were able to avoid all uncertainties in the phase diagram and its associated tie lines between Lo and Ld phases. This work combines methodology from three separate inputs: SAXS/osmotic stress experiments, comprehensive MC simulations, and detailed calculations of van der Waals interactions.

The reported values for fundamental surface forces and bending moduli are, to our knowledge, the first of their kind to be directly obtained from coexisting Lo/Ld domains. The underlying full *q*-range SAXS analysis allowed us to quantify the extent of fluctuations and capture their dependence on osmotic pressure, which proved essential for determining the bending rigidities of cholesterol-rich phases. We obtained bending moduli of 44 zJ for Ld and a roughly threefold higher value for Lo domains, attributable to their larger concentrations of saturated lipid and cholesterol.

Although we obtained almost identical van der Waals interactions for aligned Lo and Ld domains, the remaining interactions turned out to be strikingly different: decay lengths of the hydration pressures differed by 25% between Lo and Ld phases, and repulsions due to thermal fluctuations were found to be significantly increased for Ld. These findings provide evidence that a combination of hydration repulsion and fluctuation-driven undulation repulsion must be considered in any quantitative explanation of the long-range positional correlations between aligned Lo and Ld domains. In particular, the strong entropic contribution from undulating Ld domains may be a leading term to be considered. We therefore expect that our study will form the basis for a concise theory of domain alignment.

## Author Contributions

B.K. designed and performed research, analyzed data, and wrote the article; P.H. designed and performed research and analyzed data; R.P. and J.F.N. contributed analytic tools and wrote the article; G.P. designed and performed research and wrote the article.

## Figures and Tables

**Table 1:** Optimal parameters for describing the coexisting Lo/Ld phases

	Ld	Lo
*K*_c_/zJ	44 ± 10	120 ± 20
*A*/Pa	10^8.3±0.2^	10^8.1±0.2^
*λ*/Å	1.37 ± 0.15	1.74 ± 0.15
$\chi_{\text{red}}^{2}$	1.5 ± 0.5	5.8 ± 0.5

The mixture was DOPC/DSPC/Chol (0.42:0.37:0.21). Errors are reported as obtained from the fitting routine (see text for further details).

## Supporting Material

Document S1. Supporting Materials and Methods, five figures, and one table

Document S2. Article plus Supporting Material

## Supporting Citations

References (84–93) appear in the Supporting Material.
