# Supplementary material for: Bending Rigidities and Interdomain Forces in Membranes with Coexisting Lipid Domains
Source: Biophys J. 2015 Jun 16;108(12):2833–42. doi: 10.1016/j.bpj.2015.05.003 (PMC4472082; doi:10.1016/j.bpj.2015.05.003)
Supplement: Document S1. Supporting Materials and Methods, five figures, and one table [file mmc1.pdf]

Supporting information for: Bending rigidities and interdomain forces in membranes with coexisting lipid domains

B. Kollmitzer, P. Heftberger, R. Podgornik,
J. F. Nagle, and G. Pabst

April 13, 2015, Graz

S1 Area extension modulus estimation

The dependence of bilayer thickness on osmotic pressure P is accounted for via the area extension modulus K_A and given by the equation[1]

$$d_B(P) = d_B(0)\frac{K_A + P\,d(P)}{K_A + P\,d_B(0)}. \tag{S1}$$

We estimated K_A for our coexisting liquid phases based on published data for single lipids and binary lipid mixtures by Rawicz *et al.*[2,3] The Ld phase under investigation consists essentially of DOPC, with approximately 10 mol% cholesterol.[4] Interpolating linearly between the two published values for 0 and 50 mol% cholesterol in DOPC[2] yields $K_A(\mathrm{Ld}) = (430 \pm 30)\,\mathrm{mN\,m^{-1}}$.

In the coexisting Lo phase, the main constituent is the saturated lipid DSPC, which is accompanied by ca. 30 mol% cholesterol.[4] Unfortunately, published K_A values for saturated lipids are sparse. As a compromise, we interpolated linearly between pure DMPC (0 mol% cholesterol) and a 1:1 mixture of sphingomyelin/cholesterol,[2,3] yielding $K_A = (2100 \pm 500)\,\mathrm{mN\,m^{-1}}$ for our Lo phase.

As pointed out in the section *X-ray measurements* of the main text, knowing the magnitude of K_A is more important than getting the precise number. That is because the biggest estimated change in bilayer thickness turned out to be just 0.3 Å. In principle, such a subtle difference in d_B would be resolvable with SAXS, but not with the additional scattering signal due to PEG.

S2 Finite size convergence

With open edges, one generally expects a 'surface' perturbation proportional to the relative size of the boundary to the interior, i.e. proportional to $1/N$ for our systems. As is

well known, periodic boundary conditions generally reduce this perturbation. They also speed up the convergence with system size, from $1/N$ to $1/N^2$ in a case well documented by Bonner and Fisher[5] (note their Fig. 1) and in the case of the one-dimensional Ising model the convergence is exponentially fast with periodic boundary condions. While another case with very slow convergence is known,[6] that one is due to very long range interactions not present in our membrane stacks. For periodic boundary conditions, the exact solution of a harmonic approximation to Eq. (3) suggests that d_W and Δ converge asymptotically like $y(N) \sim c_\infty - c_2/N^2$, i.e. convergence is expected to be faster than $1/N$ and, in agreement with the previous simulations,[7] our results are consistent with a dominant $1/N^2$ asymptotic convergence, allowing, of course, for higher order terms.

We perform simulations for several 'densities' $N \in \{N_{min}, \dots, N_{max}\}$ and fit them with the function $y(N) = c_\infty + \sum_{k=2}^{k_{max}} c_k/N^k$. Together with the originally proposed $k_{max} = 3$ and $N \in \{6, \dots, 32\}$, this method yields sufficiently precise continuum estimators c_∞, compared to the experimental uncertainties.[8] However, we found that varying the arbitrary parameters k_{max} and N_{min} influenced the final estimator stronger for some simulations (e.g. high pressures) than for others. To obtain more reliable uncertainties and perhaps even better continuum estimates, we perform now several extrapolations, with different values for k_{max} and N_{min}, but always using the highest possible N_{max}. By not changing N_{max}, we weight the most significant simulations (with the highest density) stronger. This procedure yields a list of results for $c_{\infty,l}$, which we average for the final estimator. Its uncertainty is then determined by the individual errors of $c_{\infty,l}$ (statistical uncertainty of observables due to finite simulation length) and their standard deviation (error due to finite simulation density). This procedure is closely related to the Jackknife technique.[9,10]

Comparisons between these improved Jackknife estimators and estimators obtained by the original method are given in Fig. S1. The relative difference in the estimators were less than 5% for all performed simulations, but most importantly, Jackknife produces a meaningful uncertainty.

S3 Efficient differentiation

A single simulation of a particular set of parameters $\vec{\Lambda} = (P, A, H, \lambda, K_c, \dots)$ contains more information in the generated time series, than the aforementioned observables which are determined by averaging. By reweighting the simulated histogram of density of states, it is possible to compute these quantities over a certain range of simulation parameters and thereby also derive their gradients.[11–13 14] This well recognized method was briefly mentioned for membrane MC simulations,[15] but has not been implemented for them previously.

We calculated the expectation value of an observable $f(u, \bar{a})$ for a different set of parameters $\vec{\Lambda}'$ from a simulation performed at $\vec{\Lambda}$ by

$$\langle f \rangle_{\vec{\Lambda}'} = \frac{\sum f_{\vec{\Lambda}'}(u, \bar{a}) \cdot \exp\left(-\delta G/kT\right)}{\sum \exp\left(-\delta G/kT\right)}, \tag{S2}$$

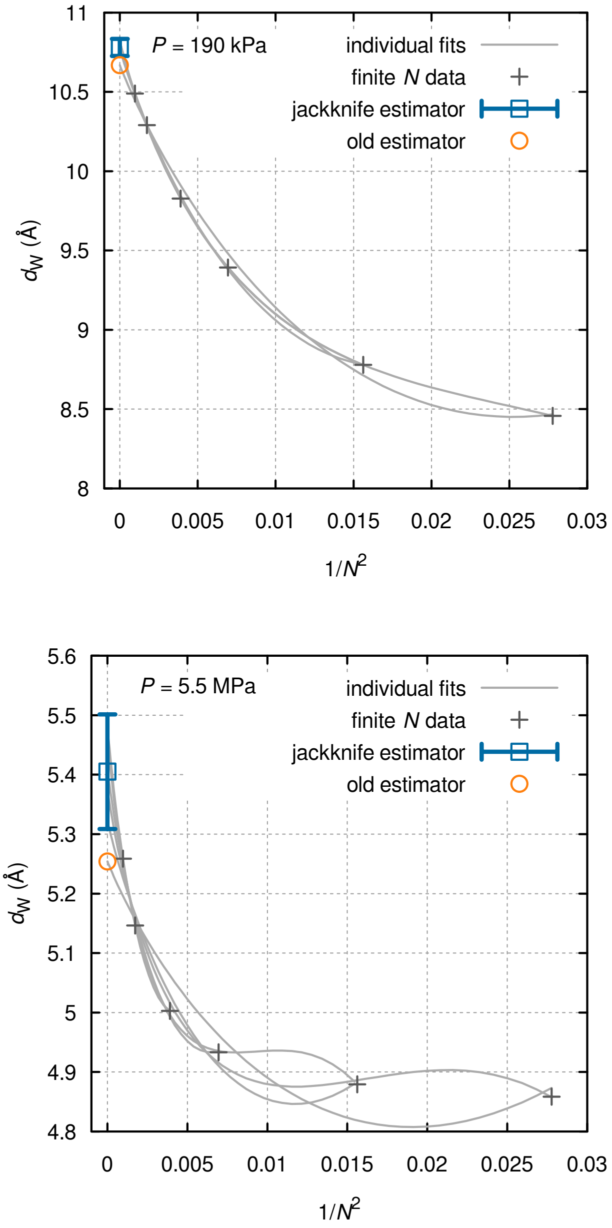

Figure S1: Finite size convergence of membrane spacing d_W vs membrane "density" N of Ld domains according to Tab. 1 at intermediate (top) and high osmotic pressures P (bottom). A variant of Jackknife allows us to obtain reasonable errors for the estimator. Statistical uncertainties for plotted finite N data are less than 10^{-2} Å.

where the sums extend over all realized configurations and δG is the change in the Gibbs energy of each state $(u, \bar{a})$ upon changing $\vec{\Lambda}$ to $\vec{\Lambda}'$. Most parameters could be separated from u and $\bar{a}$ in our case, yielding $\delta G(u, \bar{a}) = \delta\Lambda \cdot \xi(u, \bar{a})$. This allowed us to store only the time series of ξ instead of all realized states. The parameters P, A, H, and K_c were separable in this way, yielding

$$\frac{\delta G}{V} = \delta P\,\xi_P + \delta A\,\lambda\,\xi_A - \frac{\delta H}{12\pi}\,\xi_H + N^2\frac{\delta K_c}{2}\,\xi_{Kc}, \tag{S3}$$

where $\xi_P = \bar{a}/d_W$, $\xi_A = \overline{\exp(-a/\lambda)}$, $\xi_H = \overline{1/a^2}$, and $\xi_{Kc} = \overline{q^4|u_m(q_x, q_y)|^2}$. The local distance between membranes is denoted by $a = u_{m+1}(x, y) - u_m(x, y) + \bar{a}$, while the bars denote averages over (m, x, y) or (m, q_x, q_y). $V = L^2 M \bar{a}$ is the membrane stack's volume.

Separating λ from $(u, \bar{a})$ in δG turned out to be impossible, but we were able to calculate gradients of d_W and Δ with respect to λ efficiently. Because d_W and Δ did not depend explicitly on λ (i.e. $\partial f/\partial\lambda = 0$), differentiating Eq. (S2) yielded,

$$\left.\frac{\partial\langle f\rangle_{\lambda'}}{\partial\lambda'}\right|_{\lambda'=\lambda} = -\frac{AV}{kTN^2\Omega}\left(\sum f(u, \bar{a})\xi_\lambda - \langle f\rangle_\lambda \sum \xi_\lambda\right), \tag{S4}$$

where sums extend over all realized states, Ω denotes the collection length and

$$\xi_\lambda = \overline{\left(1 + \frac{a}{\lambda}\right)\exp\left(-\frac{a}{\lambda}\right)}. \tag{S5}$$

Up to first order, $\langle f\rangle_{\lambda'}$ was then determined from $\langle f\rangle_{\lambda'} \approx \langle f\rangle_\lambda + (\lambda' - \lambda)\,\partial\langle f\rangle/\partial\lambda$.

Thus, for any observable $f \in \{d_W, \Delta\}$ and parameter $\Lambda \in \{P, A, H, K_c, \lambda\}$, we first determined $\langle f\rangle_{1,2}(N)$ for $\Lambda_{1,2} = \Lambda \pm \delta\Lambda$ as detailed above, extrapolated these expectation values for $N \to \infty$ according to section S2, and finally calculated the finite difference quotient $\partial\langle f\rangle/\partial\Lambda \approx (\langle f\rangle_1 - \langle f\rangle_2)/2\delta\Lambda$. Relative finite differences were set to $\delta\Lambda/\Lambda = 0.03$.

We checked this method against direct numerical differentiation for a couple of reasonable parameters. Errors were always sufficiently small (well below 50%) to lead the optimization routine towards a global minimum (see the section *Optimizing parameters against experimental data* of the main text).

S4 Results for a homogeneous control sample

We tested our analysis on already published SAXS data for homogeneous DMPC MLVs determined at 30 °C.[16] The Lifshitz calculation of the van der Waals forces yielded a value of $H = 4.11$ zJ for the published bilayer thickness of 44.0 Å. The obtained values describing the intersurface forces are given in Tab. S1, while Fig. S2 compares the simulations with the experimental data. Reassuringly, the simulations fit the experimental osmotic pressure data well. While the fit to Δ is excellent for high hydration, the fit becomes relatively poor for Δ as d_W becomes small, similarly to our L_o sample and likely for the same reason given in the main text.

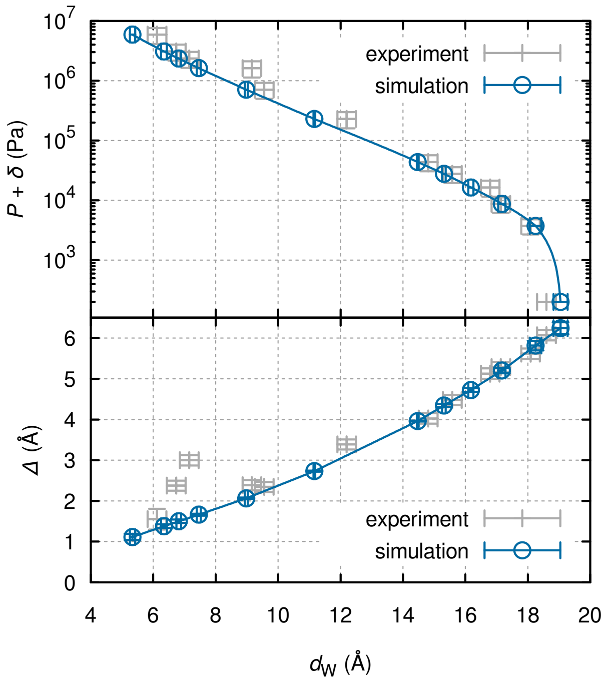

Figure S2: Osmotic pressure (top) and fluctuations (bottom) vs water-layer thickness for best fit of membrane MC simulation (cyan) against SAXS data (light gray) obtained from Ref. 16.[17] Solid lines were obtained by exponentially interpolating fluctuation contributions.

The interaction parameters obtained from the fit are shown in Tab. S1. Literature values for DMPC's bending modulus range from 50–130 zJ at 30 °C.[18] In light of this large variation, comparing only results of related methods is appropriate. Ref. 16 could not determine K_c and the modulus B separately and therefore considered several values of K_c; two of these are shown in Tab. S1. The values of A agree very well with ours. The larger values of λ would have been smaller if the true value of K_A had been known at that time. Two differences from the previous analyses are that here we calculated H and we used simulations; these cause the main differences reflected in the pairs of values for H and K_c in Tab. S1. Table S1 also shows results from another study,[19] that employed the same kind of simulations used here and differed by obtaining X-ray data from oriented stacks of DMPC bilayers, from which K_c was obtained directly. It also used the same P data, but failed to readjust the A and λ values to account for the corrected K_A. Nevertheless, agreement is reasonable.

Table S1: Optimal parameters found for describing the DMPC data published in Ref. 16.

	Current	1998a[16]	1998b[16]	2005[19]
H/zJ	4.11	7.13	4.91	6.1
K_c/zJ	57 ± 5	50	80	69
A/Pa	$10^{8.1 \pm 0.2}$	$10^{8.1}$	$10^{8.1}$	$10^{8.1}$
λ/Å	1.66 ± 0.15	1.91	1.97	1.91

For completeness, the functional dependence of the individual fundamental surface forces for DMPC is plotted in Fig. S3. The fluctuation force becomes the dominant repulsive force when d_W exceeds 9 Å, intermediate between the values of the Ld and Lo phases in Fig. 7, suggesting that the DMPC bilayer fluctuations are intermediate in this regard between the more fluid Ld phase and the more ordered Lo phase in the studied mixture. This is consistent with the Ld phase having a high concentration of the more disordered unsaturated lipids and the Lo phase having longer saturated chains with cholesterol.

S5 SAXS analysis

Comparisons between full q-range SAXS analyses and experimental data are shown in Fig. S4. Deviations between data and fits, especially for higher q ranges, are due to imperfect background subtraction, as explained in the section *X-ray measurements* in the main text.

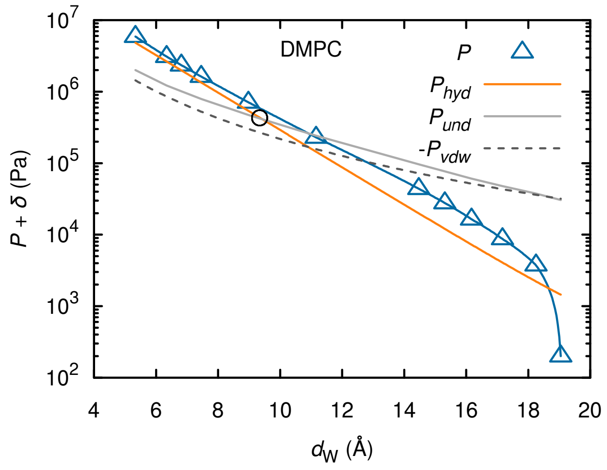

Figure S3: Partitioning of total pressure P into contributions from hydration P_{hyd}, van der Waals P_{vdw}, and undulations P_{und} for DMPC.[17] The large open black circle shows the value of the separation d_W at which hydration and undulation pressure are equal.

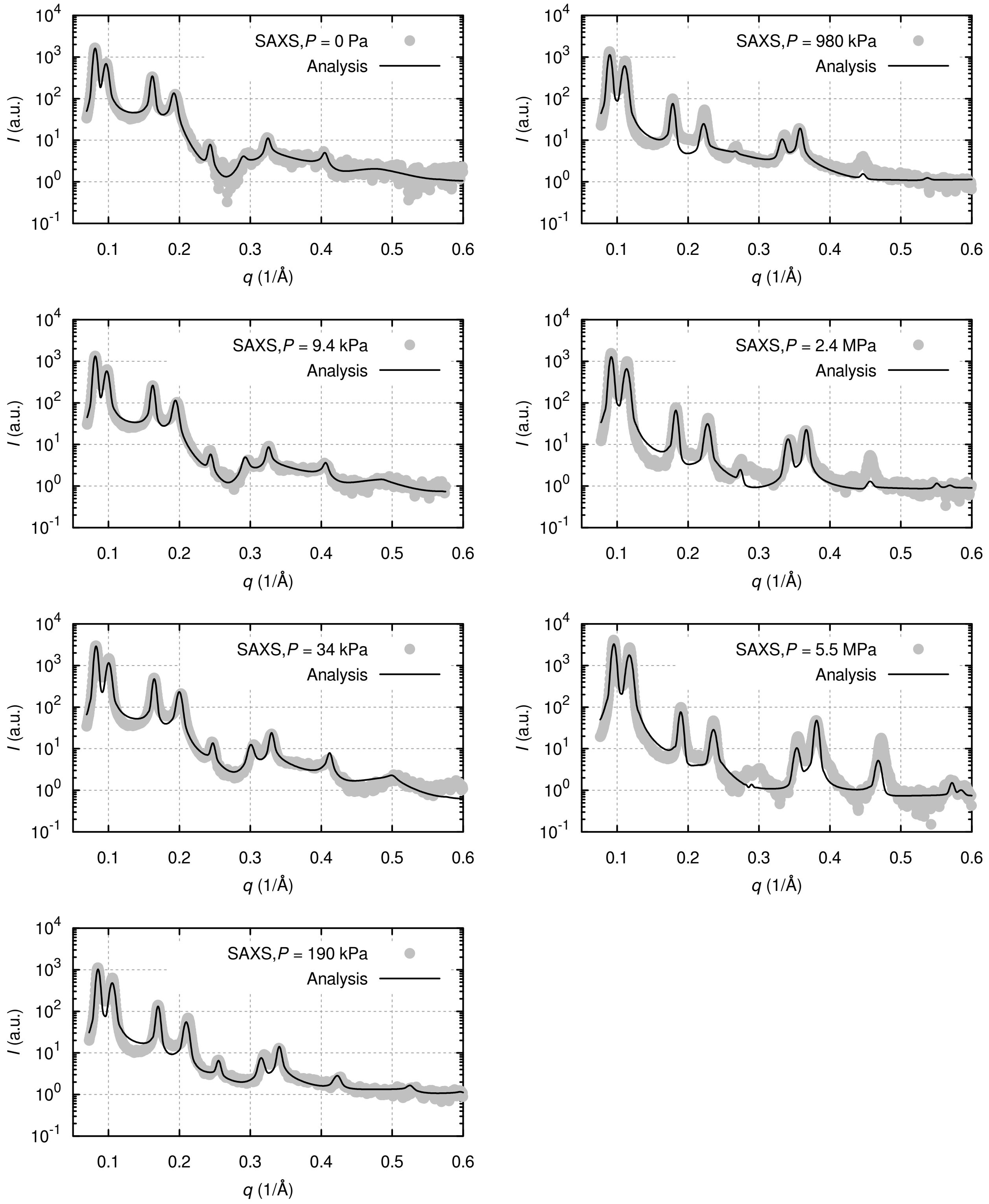

Figure S4: Calculated scattering intensities (solid lines) from full q-range analyses, compared to recorded SAXS data from coexisting phases (dots) of DOPC/DSPC/Chol (0.42:0.37:0.21), for all recorded osmotic pressures P.

S6 Fluctuations of the interbilayer water spacing

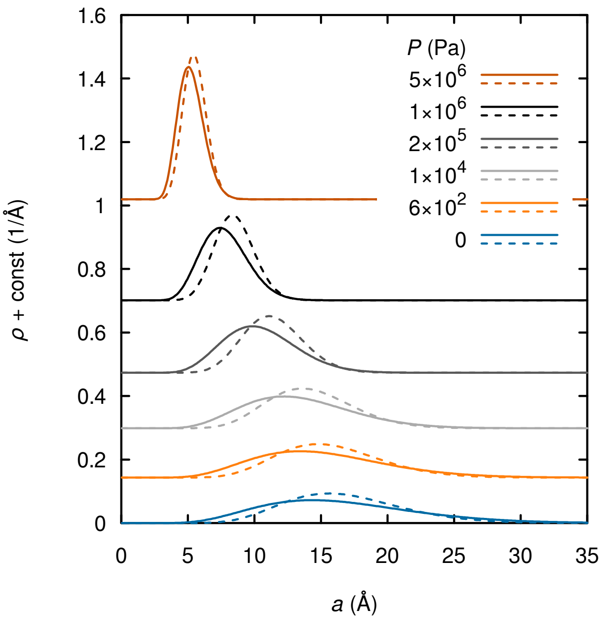

Figure S5: Probability density function ρ of the water spacing a at different external pressures P, for Ld (solid) and Lo (dashed) according to Tab. 1, obtained from $N = 32$ simulations.

References

[1] R. P. Rand and V. A. Parsegian. Hydration Forces Between Phospholipid-Bilayers. *Biochim. Biophys. Acta*, 988(3):351–376, December 1989.

[2] W. Rawicz, B.A. Smith, T.J. McIntosh, S.A. Simon, and E. Evans. Elasticity, Strength, and Water Permeability of Bilayers that Contain Raft Microdomain-Forming Lipids. *Biophys. J.*, 94(12):4725–4736, June 2008.

[3] W. Rawicz, K. C. Olbrich, T. McIntosh, D. Needham, and E. Evans. Effect of Chain Length and Unsaturation on Elasticity of Lipid Bilayers. *Biophys. J.*, 79(1):328–339, July 2000.

[4] Frederick A. Heberle, Jing Wu, Shih Lin Goh, Robin S. Petruzielo, and Gerald W. Feigenson. Comparison of Three Ternary Lipid Bilayer Mixtures: FRET and ESR Reveal Nanodomains. *Biophys. J.*, 99(10):3309–3318, November 2010.

[5] Jill C. Bonner and Michael E. Fisher. Linear Magnetic Chains with Anisotropic Coupling. *Phys. Rev.*, 135(3A):A640–A658, August 1964.

[6] J. F. Nagle and J. C. Bonner. Numerical studies of the Ising chain with long-range ferromagnetic interactions. *J. Phys. C: Solid State Phys.*, 3(2):352, February 1970.

[7] Nikolai Gouliaev and John F. Nagle. Simulations of Interacting Membranes in the Soft Confinement Regime. *Phys. Rev. Lett.*, 81(12):2610–2613, September 1998.

[8] Nikolai Gouliaev and John F. Nagle. Simulations of a single membrane between two walls using a Monte Carlo method. *Phys. Rev. E*, 58(1):881–888, July 1998.

[9] M. H. Quenouille. Notes on Bias in Estimation. *Biometrika*, 43(3/4):353–360, December 1956.

[10] John W. Tukey. Bias and confidence in not quite large samples (abstract). *Ann. Math. Statist.*, 29(2):614, June 1958.

[11] Z. W. Salsburg, J. D. Jacobson, W. Fickett, and W. W. Wood. Application of the Monte Carlo Method to the Lattice-Gas Model. I. Two-Dimensional Triangular Lattice. *J. Chem. Phys.*, 30(1):65–72, January 1959.

[12] Alan M. Ferrenberg and Robert H. Swendsen. New Monte Carlo technique for studying phase transitions. *Phys. Rev. Lett.*, 61(23):2635–2638, December 1988.

[13] Alan M. Ferrenberg and Robert H. Swendsen. Optimized Monte Carlo data analysis. *Phys. Rev. Lett.*, 63(12):1195–1198, September 1989.

[14] A common extension of this method would even allow to combine information from multiple simulations (WHAM)[20].

[15] Nikolai Gouliaev. *Monte-Carlo simulations of membrane systems.* PhD thesis, Carnegie Mellon University, Pittsburgh, Pennsylvania, USA, July 1998.

[16] Horia I. Petrache, Nikolai Gouliaev, Stephanie Tristram-Nagle, Ruitian Zhang, Robert M. Suter, and John F. Nagle. Interbilayer interactions from high-resolution x-ray scattering. *Phys. Rev. E*, 57(6):7014–7024, June 1998.

[17] The small offset $\delta = 200\,\mathrm{Pa}$ is necessary for plotting $P = 0$.

[18] John F. Nagle. Introductory Lecture: Basic quantities in model biomembranes. *Faraday Discuss.*, 161:11, 2013.

[19] Nanjun Chu, Norbert Kučerka, Yufeng Liu, Stephanie Tristram-Nagle, and John F. Nagle. Anomalous swelling of lipid bilayer stacks is caused by softening of the bending modulus. *Phys. Rev. E*, 71(4):041904, April 2005.

[20] Shankar Kumar, John M. Rosenberg, Djamal Bouzida, Robert H. Swendsen, and Peter A. Kollman. The weighted histogram analysis method for free-energy calculations on biomolecules. I. The method. *J. Comput. Chem.*, 13(8):1011–1021, 1992.
