# Supplementary material for: Bending Rigidities and Interdomain Forces in Membranes with Coexisting Lipid Domains
Source: Biophys J. 2015 Jun 16;108(12):2833–42. doi: 10.1016/j.bpj.2015.05.003 (PMC4472082; doi:10.1016/j.bpj.2015.05.003)
Supplement: Document S2. Article plus Supporting Material [file mmc2.pdf]

Biophysical Journal Volume 108 June 2015 2833–2842

Article

Bending Rigidities and Interdomain Forces in Membranes with Coexisting Lipid Domains

Benjamin Kollmitzer,[1,2] Peter Heftberger,[1,2] Rudolf Podgornik,[3,4,5] John F. Nagle,[6] and Georg Pabst[1,2,*]
[1]University of Graz, Institute of Molecular Biosciences, Biophysics Division, NAWI Graz, Graz, Austria; [2]BioTechMed-Graz, Graz, Austria; [3]Department of Theoretical Physics, Jožef Stefan Institute, Ljubljana, Slovenia; [4]Department of Physics, Faculty of Mathematics and Physics, University of Ljubljana, Ljubljana, Slovenia; [5]Department of Physics, University of Massachusetts, Amherst, Massachusetts; and [6]Department of Physics, Carnegie Mellon University, Pittsburgh, Pennsylvania

ABSTRACT To precisely quantify the fundamental interactions between heterogeneous lipid membranes with coexisting liquid-ordered (Lo) and liquid-disordered (Ld) domains, we performed detailed osmotic stress small-angle x-ray scattering experiments by exploiting the domain alignment in raft-mimicking lipid multibilayers. Performing a Monte Carlo-based analysis allowed us to determine with high reliability the magnitude and functional dependence of interdomain forces concurrently with the bending elasticity moduli. In contrast to previous methodologies, this approach enabled us to consider the entropic undulation repulsions on a fundamental level, without having to take recourse to crudely justified mean-field-like additivity assumptions. Our detailed Hamaker-coefficient calculations indicated only small differences in the van der Waals attractions of coexisting Lo and Ld phases. In contrast, the repulsive hydration and undulation interactions differed significantly, with the latter dominating the overall repulsions in the Ld phase. Thus, alignment of like domains in multibilayers appears to originate from both, hydration and undulation repulsions.

INTRODUCTION

Diverse physiological processes in living systems depend on fundamental physical interactions between lipid membranes acting on the nanoscopic length scale. Of particular interest in this context are, in addition to intramembrane interactions (1,2), forces acting between membrane domains/rafts across the aqueous phase, which are also involved in their correlated mutual alignment. Such positional correlations are well established for liquid-ordered (Lo)/liquid-disordered (Ld) domains in model lipid multibilayers (3–11). Several groups have established compositional phase diagrams for mixtures of high-melting lipid, low-melting lipid, and cholesterol, which exhibit Lo/Ld phase coexistence over a broad range of compositions and temperatures (12,13). These systems mimic mammalian outer plasma membranes and make it possible to study domain properties under well-defined conditions. Most recently, we reported structural details of Lo/Ld phases in two ternary lipid mixtures using a global small-angle x-ray scattering (SAXS) analysis for coexisting lipid domains (11). This analysis relies on the above-mentioned mutual alignment of like domains. However, domain alignment is also of biological relevance, for example, in the context of the immune response, where organization of receptor-ligand domains occurs during T-cell adhesion (14,15). Both the formation of such domains and the adhesion affinity depend strongly on thermal fluctuations and, consequently, on the bending rigidity of membranes (16,17). It is therefore reasonable to expect that fundamental intermembrane interactions will play an important role also in receptor-ligand domain alignment.

Within the broad Derjaguin-Landau-Verwey-Overbeek (DLVO) paradigm (18), the fundamental long-range interactions between soft material interfaces, mediated by their molecular environment, such as solvation (hydration) interaction, electrostatic interaction, and van der Waals interaction, can be treated independently and additively. However, this additivity Ansatz is in general not vindicated for entropically driven bending undulation interactions, which warrant a more sophisticated approach (18–20).

Besides the fundamental role of entropic membrane undulations, their relation with the membrane bending rigidity, K_c (19), and through it their connection with diverse physiological processes, has spurred a sustained scientific interest (21). Shape analysis of giant unilamellar vesicles (GUVs) (22), diffuse x-ray scattering from oriented lipid multibilayers (23), and GUV micropipette aspiration (24) are all techniques exploiting this connection, but so far, none of them has been able to simultaneously determine the bending moduli for coexisting membrane phases. On the other hand, macroscopically sized domains form distinct lamellar lattices in multibilayer systems, making it possible to apply osmotic stress experiments (8,25). In such experiments, osmotic pressure is maintained by, e.g., large neutral polymers, such as polyethylene glycol (PEG), which do not

Submitted February 25, 2015, and accepted for publication May 3, 2015.
*Correspondence: georg.pabst@uni-graz.at
This is an open access article under the CC BY license (http://creativecommons.org/licenses/by/4.0/).
Editor: Anne Kenworthy.
© 2015 The Authors
0006-3495/15/06/2833/10 $2.00

http://dx.doi.org/10.1016/j.bpj.2015.05.003
CrossMark

penetrate into the interbilayer water layer, whereas the corresponding bilayer separation and more recently also the specific line broadening due to fluctuations are measured by SAXS. Several groups, including ours, have previously applied this approach to study interactions between macromolecules, including lipid bilayers (8,25–34).

The bare long-range DLVO interaction components, which couple macromolecular surfaces through their molecular environment, get inextricably intertwined through the thermally driven conformational fluctuations of the soft interfaces, making detailed predictions of the overall interaction nearly impossible. Therefore, many studies in the past have resorted to describing such complicated thermal fluctuation effects by different mean-field/additivity approximations, where conformational fluctuation effects on the bare interaction potentials are included self-consistently (19,20,35–37). In contrast, additivity/mean-field approximations can be altogether avoided in the case of simulations that start from fundamental long-range DLVO interaction components and need no additional approximations to yield an accurate estimate for the total osmotic pressure in the system (38,39).

To understand the coupling between bare interactions and thermal undulations in phase-separated systems, we apply a gradient-based optimization algorithm to iteratively adjust the parameters in Monte Carlo (MC) simulations, i.e., the coefficients describing the strength and range of intermembrane interactions, as well as the bending rigidity characterizing the thermal undulations, to best match simulation results with the experimental osmotic stress data for coexisting Lo/Ld phases. We demonstrate the capability of the simulation-driven analysis choosing a well-studied mixture of dioleoyl phosphatidylcholine (DOPC), distearoyl phosphatidylcholine (DSPC), and cholesterol (Chol) (40–42), previously shown to exhibit Lo/Ld domain alignment in the phase-coexistence regime (11). We find that Lo domains are about three times more rigid than Ld domains, which exhibit significant contributions to domain repulsion from bending fluctuations. On the other hand, hydration forces decay much slower with domain separation between Lo domains. In turn, attractive van der Waals interactions were found to be of similar magnitude between Lo domains and between Ld domains. Our results provide insight into the strength and distance dependence of forces at play between like domains as a prerequisite to devising theories for domain alignment.

MATERIALS AND METHODS

Sample preparation

DSPC, DOPC, and Chol were purchased from Avanti Polar Lipids (Alabaster, AL) and used without further purification. PEG with an average molecular weight of 8000 was obtained from Fluka Chemie (Buchs, Switzerland) and used as received.

After weighing, lipids were dissolved in chloroform/methanol 2:1 at concentrations of 10 mg mL^{-1}. The supplier-provided molecular weights, accounting for an additional water molecule with DOPC, were used for determining stock concentrations. We prepared the ternary lipid-only mixture DOPC/DSPC/Chol (0.42:0.37:0.21) in a glass vial and evaporated the organic solvent under a gentle nitrogen stream at 30°C. This lipid composition and its tie line lie well inside the Lo/Ld phase-coexistence region according to Zhao et al. (40) and Heberle et al. (41), and the domains' structural properties have already been investigated by different methods (11,42). The remaining solvent traces were removed by placing the samples in a vacuum overnight. The next day, 18 MΩ cm water (UHQ PS, USF Elga, Wycombe, United Kingdom) was added at 20 μL water/mg lipid and the mixtures were fully hydrated at 50°C for 4 h with repeated freeze-thaw cycles.

To exert osmotic pressure on multilamellar lipid vesicles, samples were cooled to room temperature after hydration and aliquots were overlaid with PEG dissolved in water, yielding final concentrations of 1–42 wt% PEG in water. Samples were protected against oxidation with argon, and the vials were closed, taped, and stored at 4°C for 7–10 days prior to measurement. The osmotic equation of state for PEG, connecting its osmotic pressure with its solution concentration, is well known (43) and allows for an accurate determination of PEG osmotic pressure P by using previously reported high-resolution data (44).

X-ray measurements

SAXS was performed at the Austrian SAXS beamline at ELETTRA, Trieste, Italy (45,46), at a wavelength of 1.54 Å and an energy dispersion, $\Delta E/E$, of 2.5×10^{-3}. We used a mar300 Image Plate 2D detector (marresearch, Norderstedt, Germany) covering a q-range from 0.2 to 7.1 Å^{-1} and calibrated with silver-behenate ($CH_3(CH_2)_{20}-COOAg$) with a d-spacing of 5.838 nm (47). Samples were filled into reusable quartz-glass capillaries and kept in a brass sample holder connected to a circulating water bath (Huber, Offenburg, Germany). The samples were equilibrated for 10 min at (20.0 ± 0.1)°C before exposing them for 30 s to the x-ray beam.

The two-dimensional detector signal was radially integrated with FIT2D (48,49). Water background subtraction for samples without PEG was performed with Primus (50). For osmotically stressed samples however, additional scattering from PEG made a standard background subtraction impractical. Since the essential information in this case was the shapes and positions of the Bragg peaks, we subtracted approximate backgrounds, obtained by interpolating between SAXS signals of pure water and PEG/water mixtures. Alternatively, one could just subtract an arbitrary smooth function from the measured spectra.

The reduced data were then fitted using a recently published, full q-range analysis method for coexisting liquid/liquid membrane domains (11). We checked the x-ray analysis for coexisting phases by comparing it with PEG-free, homogeneous samples prepared at the published tie-line endpoint concentrations of 0.79:0.09:0.12 for the Ld and 0.05:0.65:0.30 for the Lo phase (41). These samples were also helpful for constraining some model details (e.g., the widths and distances between molecular subgroups composing the lipid heads) in the x-ray analysis.

For the x-ray analysis, the contribution of each individual phase is modeled with a bilayer structure and a superimposed membrane lattice. The lattice description is based on a modified Caillé theory (51,52) and therefore yields the average membrane periodicity, d, and the line shape parameter, η, which is connected to the mean-square fluctuation of the membrane spacing via $\Delta^2 = \eta d^2/\pi^2$ (32). The bilayer structure of each phase is then modeled separately via probability distributions of quasi-molecular fragments (53).

Of most importance, the full q-range analysis allowed us to quantify the magnitude of fluctuations for coexisting domains. For both phases of stress-free samples, this also yields accurate electron density profiles, from which the bilayer thickness could be obtained; but this was not possible when osmotic pressure was applied. Instead, the osmotic thickening of d_B was calculated using $d_B(P) = d_B(0) \times (K_A + P \times d(P))/(K_A + P \times d_B(0))$ (31), where the area extension modulus, K_A, was estimated from published micropipette aspiration experiments on single lipids and binary lipid mixtures (54,55), as detailed in Section S1 of the Supporting Material. The

overall analysis was rather insensitive to uncertainties in K_A, because the maximal change in bilayer thickness was only slightly larger than the uncertainty of the fit ($\pm 2\%$). The definition of the bilayer thickness, d_B, was found to be more important. In principle, one could determine optimal values of d_B via a joint fit with free MC parameters, but this problem is underdetermined and led to bizarre values of d_B for different data sets (56). Instead, we defined d_B as the distance between the remotest lipid atoms, also known as the steric bilayer thickness (29); this yielded good fits and comparable results and at the same time was directly accessible from the SAXS analysis. Specifically, we used $d_B = 2(z_{CholCH3} + \sigma_{CholCH3})$, where $z_{CholCH3}$ and $\sigma_{CholCH3}$ are the position (measured from the bilayer center) and the width, respectively, of the CH_3 groups in the lipid head choline. Within measurement accuracy, the definition used in Petrache et al. (32) yields equal values.

Membrane MC simulation

The simulation code used has been described previously in detail for a single membrane between two walls and for a stack of membranes (38,39,56). For completeness, but also to highlight our modifications, we briefly summarize its basic elements.

The system under consideration consists of a stack of M fluctuating and interacting membranes of size $L \times L$, as depicted in Fig. 1. The displacement of the mth membrane from its average plane is denoted as $u_m(x,y)$, the average distance between membranes as $\bar{a}$, and the bending rigidity as K_c. Imposing periodic boundary conditions in all directions yields the Hamiltonian of a stack of membranes:

$$\mathcal{H} = \sum_{m=0}^{M-1} \int \left(\frac{K_c}{2} \left(\nabla^2 u_m\right)^2 + \Phi(a_m) \right) \mathrm{d}x\, \mathrm{d}y, \qquad (1)$$

where Φ denotes the bare interaction potential, given here by the hydration repulsion and the van der Waals attraction, and $a_m(x,y) = u_{m+1}(x,y) - u_m(x,y) + \bar{a}$ denotes the local distance between two membranes. We furthermore require that $a_m \geq 0$, meaning that membranes cannot interpenetrate.

To reduce the degrees of freedom of the system to a finite amount, the membranes are discretized on a square $N \times N$ lattice. The simulation is performed in the constant pressure ensemble (57), which converges for this model faster than constant volume simulations (39). MC updates are proposed in $\bar{a}$ and in the complex coefficients $u_m(q_x,q_y)$ of the Fourier transformation of $u_m(x,y)$. Simulating in Fourier space allows for larger moves, thereby accelerating equilibration (39). After every MC step (MCS), which corresponds to degree-of-freedom ($N^2M + 1$) update proposals, we recentered the coordinate system to correct for small center-of-mass movement as a new feature in the calculations.

Simulations were performed for $L = 700$ Å, several different N in $\{6,8,12,16,24,32\}$, $M = 8$, equilibration lengths of 3×10^3 MCS, and collection lengths of 10^4 MCS, which typically exceeded the autocorrelation time by a factor of 100. Simulations were started with MCS sizes estimated from an approximative theory (20) and then subsequently optimized during equilibration, applying either dynamically optimized MC (DOMC), or—as a new feature—the acceptance ratio method (ARM) as a backup if DOMC fails (56,58).

Several observables can be determined from converged simulations, but the two most important quantities for comparison with SAXS experiments are the temporally and spatially averaged distance between membranes $d_W = \langle \bar{a} \rangle$ and the time average of its fluctuations,

$$\Delta^2 = \left\langle \overline{\left(z_{m+1}(x,y) - z_m(x,y) - d_B - d_W\right)^2} \right\rangle, \qquad (2)$$

where the long bar denotes spatial averaging over (m,x,y), angled brackets denote time averaging, and $z_m(x,y) = u_m(x,y) + m \times (\bar{a} + d_B)$ is the position of the mth membrane in real space. Specifically, d_W corresponds to the experimental thickness of the water layers separating the lipid bilayers, whereas Δ is related to the experimental Caillé parameter η, as discussed above.

The computed observables change significantly with N/L (38,39), so simulations were performed for a sequence of values of N and the observables were then extrapolated toward $N/L \to \infty$. Further details of this finite size convergence are given in Section S2 in the Supporting Material.

It should be emphasized that our explicit purpose of making contact with the x-ray structure factor and the interactions between bilayers requires much larger systems than can be presently envisioned either for all-atom simulations, used to obtain electron density profiles, or even for the most coarse-grained molecular simulations (59). We require M bilayers in a stack, each bilayer having a large lateral size L. It has been shown in previous work (38) that $L = 700$ Å and $M = 8$ are sufficient to obtain accuracies of 1% for d_W and Δ, and that would require ~130,000 lipids with associated water in typical molecular simulations. Apart from simulation size, the necessary timescales, which scale with the fourth power of the undulation wavelength (see pp. 77–78 in Pabst et al. (60)), render molecular dynamics simulations for that purpose unfeasible. Furthermore, to fit the

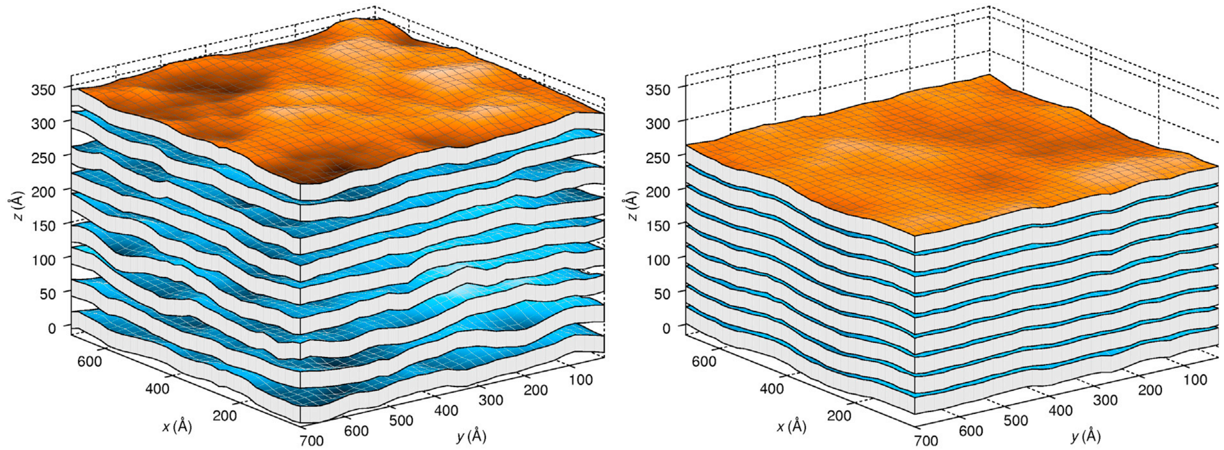

FIGURE 1 Real-space snapshots of equilibrated Ld simulations at zero (*left*) and finite (5.5 MPa) osmotic pressure (*right*). Membranes are drawn with their average thickness. Deviations from the periodic lattice are color coded. Due to 3D periodic boundary conditions, the top-most (*orange*) and bottom-most membranes are equal. The most prominent effects of external pressure, a compression of the stack and a reduction of the fluctuations, are clearly visible. To see this figure in color, go online.

experimental data requires on the order of 100 separate simulations, distributed on multiple optimizations from different start points. In the membrane MC simulations we employ, each bilayer is reduced to a network consisting of N nodes in each of the two lateral directions, and each node has only one degree of freedom.

Bare interaction potentials

For uncharged membranes, the potential at bilayer separation a is modeled canonically by

$$\Phi(a) \simeq A\lambda \exp\left(-\frac{a}{\lambda}\right) - \frac{H}{12\pi a^2}. \quad (3)$$

The first term in Eq. 3 is the well-established empirical form of the solvent-mediated hydration interaction, which has been argued to originate from changes in various measures of order for the water structure at the membrane interface (62–64), with interaction strength A and decay length λ, which is typically in the range of 1–2 Å (32). The second term describes the ubiquitous van der Waals interaction potential for two planar semiinfinite layers, with H being the Hamaker coefficient, which in general also depends on bilayer separation a, $H = H(a)$ (see p. 15 in Parsegian (65)). This functional form is convenient because it can in fact describe the cases of either two finite-thickness layers interacting across a solvent layer or effective pairwise interactions in an infinite stack of finite-thickness layers (66). For large solvent layer thickness, the nonpairwise additive effects in the latter case become negligible and the van der Waals interaction potential for the two cases follows exactly the same separation dependence.

Due to the divergence of the van der Waals potential for $a \to 0$, the $1/a^2$ term is cut off for $a < 1$ Å (38). In experiments, the collapse of charge-neutral bilayers due to van der Waals forces is avoided by very short-range steric interactions established by McIntosh et al. (61) that occur at significantly higher osmotic pressures than those relevant for the experiments presented here (see also Fig. S5). Although we added such an additional steric repulsion of the form $A_{st}\lambda_{st}\exp(-a/\lambda_{st})$ to Eq. 3, with $A_{st} = 3.6$ GPa and $\lambda_{st} = 0.6$ Å according to (61), it proved unimportant for realistic parameters.

To calculate the Hamaker coefficient, H, ab initio, we had to approximate the lipid bilayers by pure hydrocarbon. Although this model gives only a first-order estimate for the van der Waals interactions of fluctuating lipid bilayers, it is, to our knowledge, the best available approximation in the absence of data on the dielectric response of PC lipids. Further effects of, e.g., lipid-headgroup dipolar-moment fluctuations (67), could be considered as well, but they would be important only at very small separations where hydration forces dominate and the exact form of the van der Waals interaction is irrelevant. Specifically, we calculated H for an infinite stack of hydrocarbon layers in water, based on a full multilayer Lifshitz formulation (66). The ranges for the hydrocarbon thicknesses, $d_B = 45$–60 Å, and the water spacings, $d_W = 5$–60 Å, were motivated by our experimental data. In this calculation range, differences in the Hamaker coefficient were within 10%. For our MC simulations, the exact value of H matters most when all forces are of comparable magnitude, that is, at vanishing external osmotic pressure. We therefore used the H values of 4.08×10^{-21} J = 4.08 zJ for Ld and 4.15 zJ for Lo domains (see Fig. 2).

Both components of the bare potential, i.e., hydration and van der Waals interactions, cause partial bare pressures between neighboring membranes given by

$$P_{hyd}(d_W) = A\exp\left(-\frac{d_W}{\lambda}\right), \; P_{vdW}(d_W) = -\frac{H}{6\pi d_W^3}. \quad (4)$$

Equation 4 was derived from $P_j(d_W) \approx -\partial\Phi_j(d_W)/\partial d_W$. The difference from the exact relationship, $P_j(d_W) = \langle -\partial\Phi(\bar{a})/\partial\bar{a}\rangle$, was found to be less than the simulational uncertainty. For comparison to previous reports using mean-field/additivity approximations for modeling undulation interactions, one can obtain an effective decay constant λ_{und} by subtracting bare contributions from experimental data, i.e., $P_{und} = P - P_{hyd} - P_{vdW}$ (39). The undulation decay constant then results from a fit of $P_{und} = A_{und}\exp(-d_W/\lambda_{und})$, with the two adjustable parameters A_{und} and λ_{und}. Because the undulation pressure deviated significantly from a perfect exponential, we limited the fit to large separations ($d_W \geq 14$ Å).

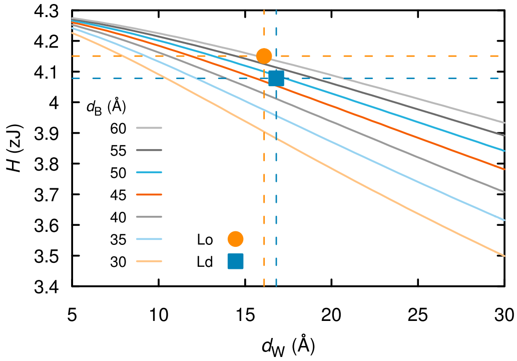

FIGURE 2 Hamaker coefficient, H, for hydrocarbon multilayers of height d_B and separation d_W in water. Highlighted are the applied values of H for Ld and Lo, which are described in the main text. To see this figure in color, go online.

Optimizing parameters against experimental data

Calculation of the Hamaker coefficient H, as described above, allowed us to reduce the number of free-fitting parameters for the simulations to three, $\vec{\Lambda} = (A, \lambda, K_c)$, for a joint analysis of domain separation and fluctuation data (see below).

We implemented a least-squares routine with Matlab (68), utilizing its trust-region reflective optimization algorithm to minimize the sum of the squared residues

$$\chi^2(\vec{\Lambda}) = \sum_i \left(\frac{d_{W,i} - d_W(P_i;\vec{\Lambda})}{U_{eff}(d_{W,i})}\right)^2 + \left(\frac{\Delta_i - \Delta(P_i;\vec{\Lambda})}{U_{eff}(\Delta_i)}\right)^2, \quad (5)$$

where $d_{W,i}$ and Δ_i are the experimentally determined values at fixed osmotic pressure P_i, $d_W(P_i;\vec{\Lambda})$ and $\Delta(P_i;\vec{\Lambda})$ are simulation results, and $U_{eff}(f)$ is the effective uncertainty of a given quantity f, derived from

$$U_{eff}^2(f) = U^2(f_{exp}) + U^2(f_{sim}) + \left(\frac{\partial f_{sim}}{\partial P} \times U(P_i)\right)^2. \quad (6)$$

The agreement between model and data was evaluated by the reduced χ^2 value, $\chi^2_{red} = \chi^2/\tilde{N}$, where $\tilde{N}$ equals the number of data points minus the number of free parameters (see p. 268 of Taylor (69)). The Jacobian for this gradient-based algorithm and the derivative in Eq. 6 were computed with the histogram reweighting method described in Section S3 in the Supporting Material. Once the iteration converged, the uncertainties of the fit parameters were determined from the curvature of χ^2_{red}. To locate the global optimum, several iterations from randomly chosen initial parameter sets were performed.

To test our implementation, we fitted simulation results determined for one reasonable parameter set, $\vec{\Lambda}'$, by starting the least squares from several different initial starting points $\vec{\Lambda}$. Within three to five iterations, these optimizations converged toward the correct values, $\vec{\Lambda}'$, thereby indicating that the weighted-histogram-based differentiation and the fit were correctly implemented. For the experimental data sets, convergence was usually reached within 10 iterations. However, due to the stochastic nature of the simulations and the consequential randomness of results and derivatives,

the optimization algorithm propagated poorly in flat regions, i.e., small $\vec{\nabla}\chi^2_{\mathrm{red}}$. Because $\chi^2_{\mathrm{red}}(\vec{\Lambda})$ is a smooth function and its gradient has to vanish at extrema, the efficiency of the optimization algorithm decreased the closer it got to the optimum. This was another reason for starting several independent iterations. Alternatively, one could have used optimization algorithms specialized for simulations (70–73), but the existing implementations did not satisfy our needs.

As a further test case, we reanalyzed previously published osmotic pressure data of pure dimyristoyl phosphocholine (DMPC) bilayers (32), yielding very reasonable values and a good agreement between simulations and experiments. Details are given in Section S4 in the Supporting Material. Thus, we conclude that our method provides a robust analysis for interactions in fluctuating membrane assemblies.

RESULTS AND DISCUSSION

X-ray analysis

SAXS patterns were analyzed as detailed previously by a Caillé theory-based analysis (11). Fig. 3 showcases the analysis for two samples at osmotic pressures of 34 kPa and 2.4 MPa, demonstrating that shapes and positions of Bragg reflections are well reproduced. Consistent with previous studies (8,9,11), we find sharper and more prominent Bragg reflections for the Lo phase due to its decreased bending fluctuations, compared to the coexisting Ld phase. Fits for all other samples are shown in Section S5 in the Supporting Material. For increased osmotic pressures, Bragg peaks shifted toward higher q and became more prominent. This is due to the decrease of bilayer separation associated with a reduction of bending fluctuations, in agreement with previous reports (32,74).

Peak line shapes for Lo and Ld domains were found to be well described by the applied Caillé theory, particular at low osmotic pressure (Fig. S4). Since this theory is incapable of fitting peaks from lamellar gel phases (75), we conclude that neither peaks assigned to the Lo nor those assigned to the Ld phase can originate from a gel phase. This is also consistent with compositional DSPC/DOPC/Chol phase diagrams reported in the literature (40,41) and a recent SAXS study from our lab, which reported for the identical lipid mixture that the structural parameters match those of Lo and Ld phases at the tie-line endpoints (11).

Fit quality of SAXS spectra worsened for increased PEG concentrations (see Fig. 3 or Section S5 in the Supporting Material). The underlying Caillé theory probably loses its applicability for the increased order experienced at elevated osmotic pressures. Although the effects on domain separation were negligible, fluctuations determined from the fits became increasingly skewed with osmotic pressure, in particular for Lo domains (see below).

The effect of osmotic pressure on the lamellar repeat spacing, d, as determined from the SAXS analysis, is plotted in Fig. 4. At high osmotic stress, the distance between bilayers is effectively set by the repulsive hydration interaction, which dominates the repulsive fluctuation interaction and the attractive van der Waals interaction. As osmotic pressure is decreased, the water spacing between bilayers, d_{W}, increases and the fluctuation interaction eventually dominates the hydration interaction. As the osmotic pressure is reduced to zero, the attractive van der Waals force balances the total repulsive forces, resulting in finite d_{W} and d values.

Within experimental uncertainty, the two isotherms in Fig. 4 are rather similar when the difference in membrane thickness, determined by $d_{\mathrm{B}}^{\mathrm{Ld}} = (48.5 \pm 1.0)\,\mathrm{\AA}$ and $d_{\mathrm{B}}^{\mathrm{Lo}} = (61.3 \pm 1.2)\,\mathrm{\AA}$, is considered. Of course, identical isotherms would imply that all the interactions are identical. However, significant experimental differences were observed in the fluctuation behavior, as detailed below, corroborating the crucial advantage of jointly analyzing fluctuations and osmotic pressure isotherms to obtain the interaction parameters (32).

Optimized simulations

The experimental data and the results of optimized simulations are compared in Fig. 5, and Table 1 lists results for the interaction parameters. Experimental errors for d_{W} and η were obtained from the SAXS analysis and those for P

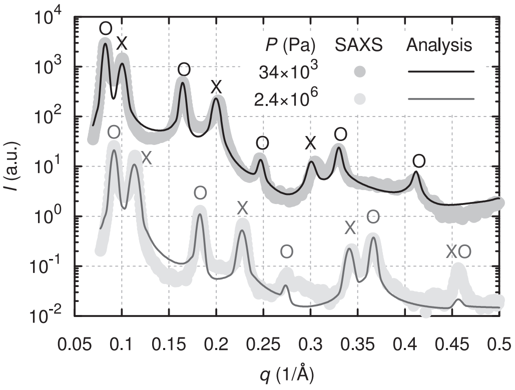

FIGURE 3 Calculated scattering intensities (*solid lines*) from full q-range analyses, compared with recorded SAXS data from coexisting phases (*dots*) for two different osmotic pressures, P. Bragg reflections from aligned Lo and Ld domains are indicated by symbols O and X, respectively.

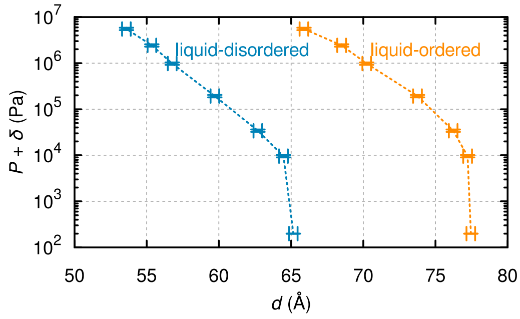

FIGURE 4 Osmotic pressure, P, versus membrane periodicity, d, for Ld and Lo determined by SAXS analysis. Dashed lines are meant solely as a guide for the eye. The small offset, $\delta = 200$ Pa, is necessary for plotting $P = 0$ on a logarithmic scale. To see this figure in color, go online.

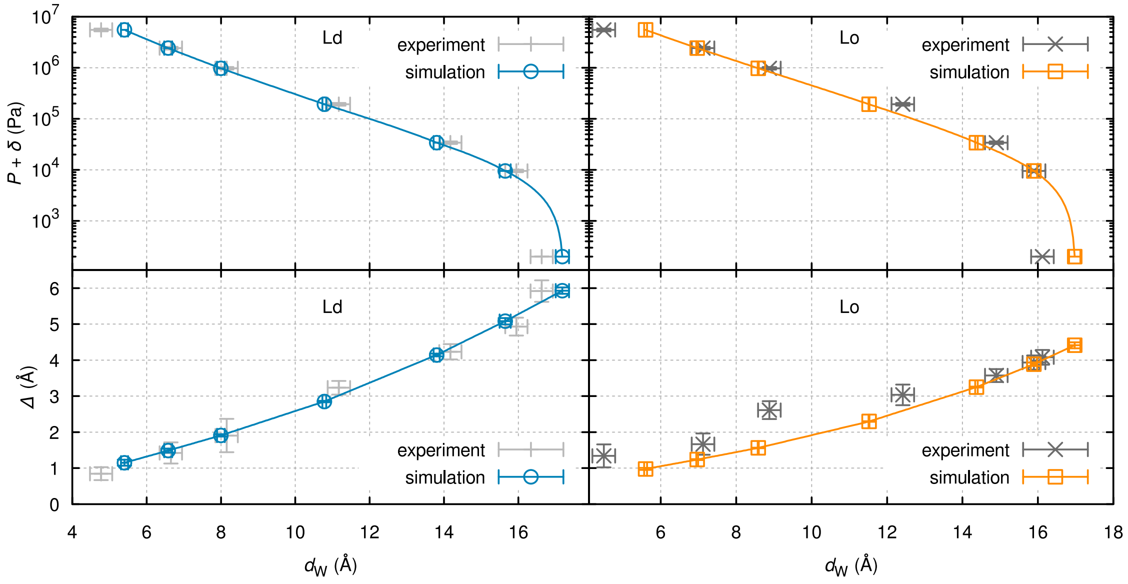

FIGURE 5 Osmotic pressure (*top*) and fluctuations (*bottom*) vs water-layer thickness for best fit of membrane MC simulation (*cyan*/*orange*) against SAXS data (*gray*). Solid lines were obtained by exponentially interpolating fluctuation contributions. The small offset, $\delta = 200$ Pa, is necessary for plotting $P = 0$ on a logarithmic scale. To see this figure in color, go online.

were estimated to equal the pipetting error of 6% for viscous PEG solutions. To quantify the agreement between data and simulations, we report $\chi^2_{\rm red}$, which becomes ~1 if the differences are compatible with experimental errors (see p. 268 in Taylor (69)). This is the case for the Ld phase, where simulations and experimental data match ideally, but the mismatch for Lo is larger than expected ($\chi^2_{\rm red} = 6$).

We are inclined to attribute this discrepancy for Lo at least partially to the limited applicability of the Caillé theory for highly ordered systems, as described in the previous section. Indeed, deviations in Δ are especially pronounced for small bilayer separations, i.e., at high osmotic pressures. In light of these discrepancies, we suggest that the experimental uncertainties determined for the Lo phase are rather too small, because they do not take into account the decreasing applicability of the Caillé theory for more ordered phases whose fluctuations are suppressed by low hydration.

Although differences in $P(d_W)$ are insignificant between Ld and Lo (see also Fig. 4), fluctuations of the Lo phase, containing most of the DSPC and about thrice as much cholesterol as Ld, are evidently smaller (Fig. 5). In the continuum mechanics treatment used in the simulations, this increase in bilayer stiffness is captured by a threefold-higher K_c for Lo (see Table 1).

The values obtained by us for K_c compare well with previously reported results obtained by different techniques. Several groups have measured the bending rigidities of binary DOPC/cholesterol mixtures, which ranged from 60 ± 8 to 100 ± 25 zJ and were found to be largely unchanged by the cholesterol content (76–79). This supports the $K_c = (44 \pm 10)$zJ obtained for Ld, where DOPC is the main constituent (41). In contrast, a larger concentration of saturated lipids, for which K_c does increase with cholesterol (76), is present in the Lo phase, so a larger bending rigidity would be expected for Lo than for Ld. Our finding of $K_c = 120 \pm 20$ zJ for the Lo phase is consistent with this expectation.

Furthermore, molecular dynamics simulation results are available for comparison. Khelashvili et al. (80) used the reported tie-line endpoint compositions (41) to separately simulate the Ld and Lo phases, obtaining bending moduli of 80–130 zJ for Ld and 340–440 zJ for Lo. Although these values are large compared to our results, both methods find a strong increase of K_c between Ld and Lo.

In agreement with Pan et al. (81), we find that a rather simple model suffices to relate bending to area extension moduli for cholesterol-rich samples (82). Based on the assumption that the main contribution to membrane rigidity comes from the stiff cholesterol ring of size δ', Pan et al. used the relationship $\delta'^2 = 12K_c/K_A$. For our samples, with $K_A = 430$ mN m^{-1} and 2100 mN m^{-1} (see Section S1 in the Supporting Material for details), this equation yields $\delta' = 11$ Å and 8 Å for Ld and Lo, respectively, in good

TABLE 1 Optimal parameters for describing the coexisting Lo/Ld phases

	Ld	Lo
K_c/zJ	44 ± 10	120 ± 20
A/Pa	$10^{8.3\pm0.2}$	$10^{8.1\pm0.2}$
λ/Å	1.37 ± 0.15	1.74 ± 0.15
$\chi^2_{\rm red}$	1.5 ± 0.5	5.8 ± 0.5

The mixture was DOPC/DSPC/Chol (0.42:0.37:0.21). Errors are reported as obtained from the fitting routine (see text for further details).

agreement with actual cholesterol ring sizes of ~9 Å, giving additional support to our analysis.

Interdomain forces

As stated previously, the differences between Ld and Lo in the P versus d_W data sets are small. However, a more thorough investigation of these quantities yields interesting insights. Because good fits to these data were obtained, the total pressure P is readily dissected into its individual contributions from the fundamental surface forces, whose functional dependences are plotted in Fig. 6.

The thicker Lo bilayer causes an increase in the Hamaker coefficient, but only by 3% compared to that of the Ld phase; this is a minor difference in the van der Waals interaction that is hardly noticeable in the P_{vdW} curve in Fig. 6. For small bilayer separations, the hydration interactions are of similar magnitude and represent, as expected, the dominant contribution to the total interaction potential for both phases. Despite these similarities, the fluctuation pressure starts to surpass the hydration pressure already at much smaller separations, d_W, for Ld than for Lo. This difference implies that, in contrast to the ordered phase, the undulation interaction becomes the most important repulsive interaction over a wider range of bilayer separations in the case of the disordered phase. Stronger repulsions due to fluctuation interactions are of course reasonable because thermal undulations were found to be significantly increased for the Ld phase (Fig. 5). Nevertheless, even in the Lo phase, the thermal undulation interaction dominates the hydration force over the most important, well hydrated range of d_W, starting at separations of 12 Å.

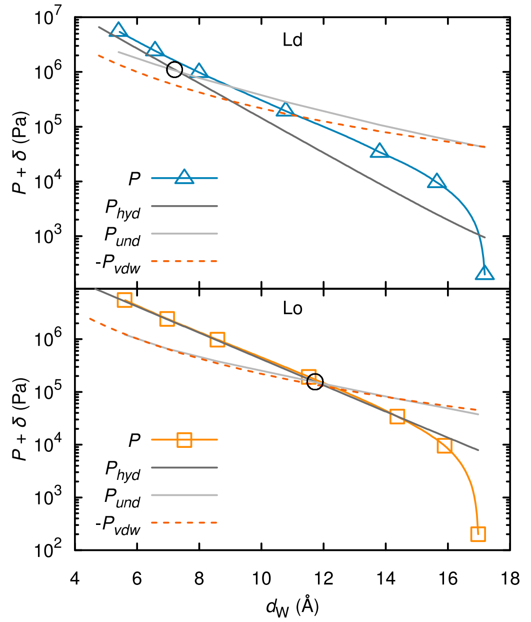

FIGURE 6 Partitioning of total pressure, P, into contributions from hydration, P_{hyd}, van der Waals, P_{vdw}, and undulation interactions, P_{und}, for Ld (*upper*) and Lo (*lower*). The large open black circles show the values of the separation, d_W, at which hydration and undulation pressure are equal. The small offset, $\delta = 200$ Pa, is necessary for plotting $P = 0$ on a logarithmic scale. δ is also responsible for the deviation of the hydration pressure from a straight line at low P. To see this figure in color, go online.

We obtained almost exponentially decreasing fluctuation forces of the scaling form $\propto \exp(-z/\lambda_{und})$, with effective decay lengths of $\lambda_{und} \approx 3.3$ Å and 3.7 Å for Ld and Lo, respectively. The ratio of fluctuation to hydration decay length λ_{und}/λ is obtained as 2.4 for Ld and 2.1 for Lo. Mean-field theory predicted this ratio's value as 2.0 (20), but values of 2.4 and 2.0–3.0 have been reported from simulations (38,39) and from other experiments (8,32,33), respectively.

Compared to Lo, a significantly shorter decay length for the hydration interaction pressure was found for the Ld phase. At present, the origin for this difference is unclear. However, it is this difference combined with the larger fluctuation force that gives P versus d_W curves that are nearly the same for Lo and Ld, both with fully hydrated d_W close to 17 Å.

Domain alignment across interlamellar aqueous phases has recently been hypothesized to be caused by water-network mismatch due to the different hydration properties of Lo and Ld phases (3). In support of this postulation, we observed significantly different hydration forces and nearly equal van der Waals forces for both phases. The aforementioned hypothesis neglected, however, influences from thermal undulation interactions, which we now find to differ considerably between coexisting Lo and Ld domains. The importance of thermal fluctuations is especially striking near full hydration, where undulation and van der Waals pressures surpass hydration repulsion by an order of magnitude (see Fig. 6).

CONCLUSIONS

We have evaluated the fundamental long-range interactions between coexisting Lo and Ld domains in DOPC/DSPC/cholesterol, which is a frequently used model system for mammalian outer plasma membranes (11–13,40–42,82,83). Because we could do this at concentrations where Lo and Ld domains coexist, we were able to avoid all uncertainties in the phase diagram and its associated tie lines between Lo and Ld phases. This work combines methodology from three separate inputs: SAXS/osmotic stress experiments, comprehensive MC simulations, and detailed calculations of van der Waals interactions.

The reported values for fundamental surface forces and bending moduli are, to our knowledge, the first of their kind to be directly obtained from coexisting Lo/Ld domains. The underlying full q-range SAXS analysis allowed us to quantify the extent of fluctuations and capture their dependence on osmotic pressure, which proved essential for determining the bending rigidities of cholesterol-rich phases. We obtained bending moduli of 44 zJ for Ld and a roughly threefold higher value for Lo domains,

attributable to their larger concentrations of saturated lipid and cholesterol.

Although we obtained almost identical van der Waals interactions for aligned Lo and Ld domains, the remaining interactions turned out to be strikingly different: decay lengths of the hydration pressures differed by 25% between Lo and Ld phases, and repulsions due to thermal fluctuations were found to be significantly increased for Ld. These findings provide evidence that a combination of hydration repulsion and fluctuation-driven undulation repulsion must be considered in any quantitative explanation of the long-range positional correlations between aligned Lo and Ld domains. In particular, the strong entropic contribution from undulating Ld domains may be a leading term to be considered. We therefore expect that our study will form the basis for a concise theory of domain alignment.

SUPPORTING MATERIAL

Supporting Materials and Methods, five figures, and one table are available at http://www.biophysj.org/biophysj/supplemental/S0006-3495(15)00461-0.

AUTHOR CONTRIBUTIONS

B.K. designed and performed research, analyzed data, and wrote the article; P.H. designed and performed research and analyzed data; R.P. and J.F.N. contributed analytic tools and wrote the article; G.P. designed and performed research and wrote the article.

ACKNOWLEDGMENTS

The computational results presented here were achieved using the Vienna Scientific Cluster (VSC). The authors thank Alexander Rieder and Heinz Amenitsch for experimental support and Hans Gerd Evertz for a critical review of the simulation and advice regarding finite size convergence.

This work was supported by the Austrian Science Fund (FWF), Project no. P24459-B20 to G.P. Support for the original development of the Monte Carlo software was provided to J.F.N. under grant GM44976 from the U.S. National Institutes of Health. R.P. acknowledges support from SLO-A bilateral grant N1-0019 of the Slovene Research Agency.

SUPPORTING CITATIONS

References (84–93) appear in the Supporting Material.

REFERENCES

1. Semrau, S., T. Idema, …, C. Storm. 2009. Membrane-mediated interactions measured using membrane domains. *Biophys. J.* 96:4906–4915.
2. Ursell, T. S., W. S. Klug, and R. Phillips. 2009. Morphology and interaction between lipid domains. *Proc. Natl. Acad. Sci. USA.* 106:13301–13306.
3. Tayebi, L., Y. Ma, …, A. N. Parikh. 2012. Long-range interlayer alignment of intralayer domains in stacked lipid bilayers. *Nat. Mater.* 11:1074–1080.
4. Karmakar, S., and V. A. Raghunathan. 2005. Structure of phospholipid-cholesterol membranes: an x-ray diffraction study. *Phys. Rev. E Stat. Nonlin. Soft Matter Phys.* 71:061924.
5. Chen, L., Z. Yu, and P. J. Quinn. 2007. The partition of cholesterol between ordered and fluid bilayers of phosphatidylcholine: a synchrotron x-ray diffraction study. *Biochim. Biophys. Acta.* 1768:2873–2881.
6. Mills, T. T., S. Tristram-Nagle, …, G. W. Feigenson. 2008. Liquid-liquid domains in bilayers detected by wide angle x-ray scattering. *Biophys. J.* 95:682–690.
7. Staneva, G., C. Chachaty, …, P. J. Quinn. 2008. The role of sphingomyelin in regulating phase coexistence in complex lipid model membranes: competition between ceramide and cholesterol. *Biochim. Biophys. Acta.* 1778:2727–2739.
8. Pabst, G., B. Boulgaropoulos, …, P. Laggner. 2009. Effect of ceramide on nonraft proteins. *J. Membr. Biol.* 231:125–132.
9. Yuan, J., A. Kiss, …, L. S. Hirst. 2009. Solution synchrotron x-ray diffraction reveals structural details of lipid domains in ternary mixtures. *Phys. Rev. E Stat. Nonlin. Soft Matter Phys.* 79:031924.
10. Uppamoochikkal, P., S. Tristram-Nagle, and J. F. Nagle. 2010. Orientation of tie-lines in the phase diagram of DOPC/DPPC/cholesterol model biomembranes. *Langmuir.* 26:17363–17368.
11. Heftberger, P., B. Kollmitzer, …, G. Pabst. 2015. In situ determination of structure and fluctuations of coexisting fluid membrane domains. *Biophys. J.* 108:854–862.
12. Heberle, F. A., and G. W. Feigenson. 2011. Phase separation in lipid membranes. *Cold Spring Harb. Perspect. Biol.* 3:a004630.
13. Marsh, D. 2009. Cholesterol-induced fluid membrane domains: a compendium of lipid-raft ternary phase diagrams. *Biochim. Biophys. Acta.* 1788:2114–2123.
14. Monks, C. R. F., B. A. Freiberg, …, A. Kupfer. 1998. Three-dimensional segregation of supramolecular activation clusters in T cells. *Nature.* 395:82–86.
15. Grakoui, A., S. K. Bromley, …, M. L. Dustin. 1999. The immunological synapse: a molecular machine controlling T cell activation. *Science.* 285:221–227.
16. Różycki, B., R. Lipowsky, and T. R. Weikl. 2010. Segregation of receptor-ligand complexes in cell adhesion zones: phase diagrams and the role of thermal membrane roughness. *New J. Phys.* 12:095003.
17. Hu, J., R. Lipowsky, and T. R. Weikl. 2013. Binding constants of membrane-anchored receptors and ligands depend strongly on the nanoscale roughness of membranes. *Proc. Natl. Acad. Sci. USA.* 110:15283–15288.
18. Israelachvili, J. N. 2011. Intermolecular and Surface Forces. Academic Press, Waltham, MA, pp. 577–616.
19. Helfrich, W. 1978. Steric interaction of fluid membranes in multilayer systems. *Z. Naturforsch.* 33:305–315.
20. Podgornik, R., and V. A. Parsegian. 1992. Thermal-mechanical fluctuations of fluid membranes in confined geometries: the case of soft confinement. *Langmuir.* 8:557–562.
21. Pabst, G. 2013. Coupling membrane elasticity and structure to protein function. *In* Advances in Planar Lipid Bilayers and Liposomes, *Volume 18* A. Iglič, and C. V. Kulkarni, editors. Elsevier, New York, pp. 81–109.
22. Méléard, P., C. Gerbeaud, …, P. Bothorel. 1997. Bending elasticities of model membranes: influences of temperature and sterol content. *Biophys. J.* 72:2616–2629.
23. Lyatskaya, Y., Y. Liu, …, J. F. Nagle. 2001. Method for obtaining structure and interactions from oriented lipid bilayers. *Phys. Rev. E Stat. Nonlin. Soft Matter Phys.* 63:011907.
24. Evans, E., and W. Rawicz. 1990. Entropy-driven tension and bending elasticity in condensed-fluid membranes. *Phys. Rev. Lett.* 64:2094–2097.
25. Boulgaropoulos, B., M. Rappolt, …, G. Pabst. 2012. Lipid sorting by ceramide and the consequences for membrane proteins. *Biophys. J.* 102:2031–2038.

26. LeNeveu, D. M., and R. P. Rand. 1977. Measurement and modification of forces between lecithin bilayers. *Biophys. J.* 18:209–230.

27. Parsegian, V. A., N. Fuller, and R. P. Rand. 1979. Measured work of deformation and repulsion of lecithin bilayers. *Proc. Natl. Acad. Sci. USA.* 76:2750–2754.

28. Parsegian, V. A., R. P. Rand, …, D. C. Rau. 1986. Osmotic stress for the direct measurement of intermolecular forces. *Methods Enzymol,.* 127:400–416.

29. McIntosh, T. J., and S. A. Simon. 1986. Hydration force and bilayer deformation: a reevaluation. *Biochemistry.* 25:4058–4066.

30. McIntosh, T. J., and S. A. Simon. 1993. Contributions of hydration and steric (entropic) pressures to the interactions between phosphatidylcholine bilayers: experiments with the subgel phase. *Biochemistry.* 32:8374–8384.

31. Rand, R. P., and V. A. Parsegian. 1989. Hydration forces between phospholipid bilayers. *Biochim. Biophys. Acta.* 988:351–376.

32. Petrache, H. I., N. Gouliaev, …, J. F. Nagle. 1998. Interbilayer interactions from high-resolution x-ray scattering. *Phys. Rev. E Stat. Phys. Plasmas Fluids Relat. Interdiscip. Topics.* 57:7014–7024.

33. Pabst, G., S. Danner, …, J. Katsaras. 2007. Entropy-driven softening of fluid lipid bilayers by alamethicin. *Langmuir.* 23:11705–11711.

34. Pabst, G., N. Kučerka, …, J. Katsaras. 2010. Applications of neutron and x-ray scattering to the study of biologically relevant model membranes. *Chem. Phys. Lipids.* 163:460–479.

35. Sornette, D., and N. Ostrowsky. 1986. Importance of membrane fluidity on bilayer interactions. *J. Chem. Phys.* 84:4062–4067.

36. Evans, E. A., and V. A. Parsegian. 1986. Thermal-mechanical fluctuations enhance repulsion between bimolecular layers. *Proc. Natl. Acad. Sci. USA.* 83:7132–7136.

37. Mecke, K. R., T. Charitat, and F. Graner. 2003. Fluctuating lipid bilayer in an arbitrary potential: theory and experimental determination of bending rigidity. *Langmuir.* 19:2080–2087.

38. Gouliaev, N., and J. F. Nagle. 1998. Simulations of interacting membranes in the soft confinement regime. *Phys. Rev. Lett.* 81:2610–2613.

39. Gouliaev, N., and J. F. Nagle. 1998. Simulations of a single membrane between two walls using a Monte Carlo method. *Phys. Rev. E Stat. Phys. Plasmas Fluids Relat. Interdiscip. Topics.* 58:881–888.

40. Zhao, J., J. Wu, …, G. W. Feigenson. 2007. Phase studies of model biomembranes: complex behavior of DSPC/DOPC/Cholesterol. *Biochim. Biophys. Acta.* 1768:2764–2776.

41. Heberle, F. A., J. Wu, …, G. W. Feigenson. 2010. Comparison of three ternary lipid bilayer mixtures: FRET and ESR reveal nanodomains. *Biophys. J.* 99:3309–3318.

42. Heberle, F. A., R. S. Petruzielo, …, J. Katsaras. 2013. Bilayer thickness mismatch controls domain size in model membranes. *J. Am. Chem. Soc.* 135:6853–6859.

43. Cohen, J. A., R. Podgornik, …, V. A. Parsegian. 2009. A phenomenological one-parameter equation of state for osmotic pressures of PEG and other neutral flexible polymers in good solvents. *J. Phys. Chem. B.* 113:3709–3714.

44. Stanley, C. B., and H. H. Strey. 2003. Measuring osmotic pressure of poly(ethylene glycol) solutions by sedimentation equilibrium ultracentrifugation. *Macromolecules.* 36:6888–6893.

45. Amenitsch, H., M. Rappolt, …, S. Bernstorff. 1998. First performance assessment of the small-angle x-ray scattering beamline at ELETTRA. *J. Synchrotron Radiat.* 5:506–508.

46. Bernstorff, S., H. Amenitsch, and P. Laggner. 1998. High-throughput asymmetric double-crystal monochromator of the SAXS beamline at ELETTRA. *J. Synchrotron Radiat.* 5:1215–1221.

47. Huang, T. C., H. Toraya, …, Y. Wu. 1993. X-ray powder diffraction analysis of silver behenate, a possible low-angle diffraction standard. *J. Appl. Crystallogr.* 26:180–184.

48. Hammersley, A. P. 1997. FIT2D: an introduction and overview. *European Synchrotron Radiation Facility Internal Report ESRF97HA02T.*

49. Hammersley, A. P., S. O. Svensson, …, D. Hausermann. 1996. Two-dimensional detector software: from real detector to idealised image or two-theta scan. *High Press. Res.* 14:235–248.

50. Konarev, P. V., V. V. Volkov, …, D. I. Svergun. 2003. PRIMUS: a Windows PC-based system for small-angle scattering data analysis. *J. Appl. Crystallogr.* 36:1277–1282.

51. Zhang, R., R. M. Suter, and J. F. Nagle. 1994. Theory of the structure factor of lipid bilayers. *Phys. Rev. E Stat. Phys. Plasmas Fluids Relat. Interdiscip. Topics.* 50:5047–5060.

52. Caillé, A. 1972. Physique cristalline: remarques sur la diffusion des rayons X dans les smectiques A. [Crystal physics: remarks on the scattering of x-rays in smectics.]. *C. R. Acad. Sci. Paris Ser. B.* 274:891–893.

53. Kučerka, N., J. F. Nagle, …, J. Katsaras. 2008. Lipid bilayer structure determined by the simultaneous analysis of neutron and x-ray scattering data. *Biophys. J.* 95:2356–2367.

54. Rawicz, W., B. A. Smith, …, E. Evans. 2008. Elasticity, strength, and water permeability of bilayers that contain raft microdomain-forming lipids. *Biophys. J.* 94:4725–4736.

55. Rawicz, W., K. C. Olbrich, …, E. Evans. 2000. Effect of chain length and unsaturation on elasticity of lipid bilayers. *Biophys. J.* 79:328–339.

56. Gouliaev, N. 1998. Monte-Carlo simulations of membrane systems. PhD thesis. Carnegie Mellon University, Pittsburgh, PA.

57. McDonald, I. 1972. NpT-ensemble Monte Carlo calculations for binary liquid mixtures. *Mol. Phys.* 23:41–58.

58. Bouzida, D., S. Kumar, and R. H. Swendsen. 1992. Efficient Monte Carlo methods for the computer simulation of biological molecules. *Phys. Rev. A.* 45:8894–8901.

59. Cooke, I. R., and M. Deserno. 2005. Solvent-free model for self-assembling fluid bilayer membranes: stabilization of the fluid phase based on broad attractive tail potentials. *J. Chem. Phys.* 123:224710.

60. Pabst, G., N. Kučerka, …, J. Katsaras. 2014. Liposomes, Lipid Bilayers and Model Membranes: From Basic Research to Application. CRC Press, Boca Raton, FL.

61. McIntosh, T. J., A. D. Magid, and S. A. Simon. 1987. Steric repulsion between phosphatidylcholine bilayers. *Biochemistry.* 26:7325–7332.

62. Marčelja, S., and N. Radić. 1976. Repulsion of interfaces due to boundary water. *Chem. Phys. Lett.* 42:129–130.

63. Kanduč, M., E. Schneck, and R. R. Netz. 2013. Hydration interaction between phospholipid membranes: insight into different measurement ensembles from atomistic molecular dynamics simulations. *Langmuir.* 29:9126–9137.

64. Kanduč, M., A. Schlaich, …, R. R. Netz. 2014. Hydration repulsion between membranes and polar surfaces: simulation approaches versus continuum theories. *Adv. Colloid Interface Sci.* 208:142–152.

65. Parsegian, V. A. 2006. Van der Waals Forces. Cambridge University Press, Cambridge, United Kingdom.

66. Podgornik, R., R. H. French, and V. A. Parsegian. 2006. Nonadditivity in van der Waals interactions within multilayers. *J. Chem. Phys.* 124:044709.

67. Podgornik, R. 1988. Solvent structure effects in dipole correlation forces. *Chem. Phys. Lett.* 144:503–508.

68. 2011. MATLAB v. 7.12 (R2011a).

69. Taylor, J. 1997. Introduction to Error Analysis, The Study of Uncertainties in Physical Measurements volume 1, 2nd ed.. University Science Books, New York.

70. Ben-Tal, A., and A. Nemirovski. 1999. Robust solutions of uncertain linear programs. *Oper. Res. Lett.* 25:1–13.

71. Ben-Tal, A., and A. Nemirovski. 2000. Robust solutions of Linear Programming problems contaminated with uncertain data. *Math. Program.* 88:411–424.

72. Fu, M. C. 2002. Feature article: optimization for simulation: theory vs. practice. *INFORMS J. Comput.* 14:192–215.

73. Ben-Tal, A., A. Goryashko, …, A. Nemirovski. 2004. Adjustable robust solutions of uncertain linear programs. *Math. Program. Ser. A.* 99:351–376.

74. Hemmerle, A., L. Malaquin, …, J. Daillant. 2012. Controlling interactions in supported bilayers from weak electrostatic repulsion to high osmotic pressure. *Proc. Natl. Acad. Sci. USA.* 109:19938–19942.

75. Pabst, G. 2006. Global properties of biomimetic membranes: perspectives on molecular features. *Biophys. Rev. Lett.* 01:57–84.

76. Pan, J., T. T. Mills, …, J. F. Nagle. 2008. Cholesterol perturbs lipid bilayers nonuniversally. *Phys. Rev. Lett.* 100:198103.

77. Sorre, B., A. Callan-Jones, …, P. Bassereau. 2009. Curvature-driven lipid sorting needs proximity to a demixing point and is aided by proteins. *Proc. Natl. Acad. Sci. USA.* 106:5622–5626.

78. Tian, A., B. R. Capraro, …, T. Baumgart. 2009. Bending stiffness depends on curvature of ternary lipid mixture tubular membranes. *Biophys. J.* 97:1636–1646.

79. Gracià, R. S., N. Bezlyepkina, …, R. Dimova. 2010. Effect of cholesterol on the rigidity of saturated and unsaturated membranes: fluctuation and electrodeformation analysis of giant vesicles. *Soft Matter.* 6:1472–1482.

80. Khelashvili, G., B. Kollmitzer, …, D. Harries. 2013. Calculating the bending modulus for multicomponent lipid membranes in different thermodynamic phases. *J. Chem. Theory Comput.* 9:3866–3871.

81. Pan, J., S. Tristram-Nagle, and J. F. Nagle. 2009. Effect of cholesterol on structural and mechanical properties of membranes depends on lipid chain saturation. *Phys. Rev. E Stat. Nonlin. Soft Matter Phys.* 80:021931.

82. Veatch, S. L., and S. L. Keller. 2003. Separation of liquid phases in giant vesicles of ternary mixtures of phospholipids and cholesterol. *Biophys. J.* 85:3074–3083.

83. Scherfeld, D., N. Kahya, and P. Schwille. 2003. Lipid dynamics and domain formation in model membranes composed of ternary mixtures of unsaturated and saturated phosphatidylcholines and cholesterol. *Biophys. J.* 85:3758–3768.

84. Salsburg, Z. W., J. D. Jacobson, …, W. W. Wood. 1959. Application of the Monte Carlo method to the lattice-gas model. I. Two-dimensional triangular lattice. *J. Chem. Phys.* 30:65–72.

85. Ferrenberg, A. M., and R. H. Swendsen. 1988. New Monte Carlo technique for studying phase transitions. *Phys. Rev. Lett.* 61:2635–2638.

86. Ferrenberg, A. M., and R. H. Swendsen. 1989. Optimized Monte Carlo data analysis. *Phys. Rev. Lett.* 63:1195–1198.

87. Kumar, S., J. M. Rosenberg, …, P. A. Kollman. 1992. The weighted histogram analysis method for free-energy calculations on biomolecules. I. The method. *J. Comput. Chem.* 13:1011–1021.

88. Nagle, J. F. 2013. Introductory lecture: basic quantities in model biomembranes. *Faraday Discuss.* 161:11–29, discussion 113–150.

89. Chu, N., N. Kučerka, …, J. F. Nagle. 2005. Anomalous swelling of lipid bilayer stacks is caused by softening of the bending modulus. *Phys. Rev. E Stat. Nonlin. Soft Matter Phys.* 71:041904.

90. Bonner, J. C., and M. E. Fisher. 1964. Linear magnetic chains with anisotropic coupling. *Phys. Rev.* 135:A640–A658.

91. Nagle, J. F., and J. C. Bonner. 1970. Numerical studies of the Ising chain with long-range ferromagnetic interactions. *J. Phys. C Solid State Phys.* 3:352–366.

92. Quenouille, M. H. 1956. Notes on bias in estimation. *Biometrika.* 43:353–360.

93. Tukey, J. W. 1958. Bias and confidence in not quite large samples. *Ann. Math. Stat.*: Abstract, 29:614.

Supporting information for: Bending rigidities and interdomain forces in membranes with coexisting lipid domains

B. Kollmitzer, P. Heftberger, R. Podgornik,
J. F. Nagle, and G. Pabst

April 13, 2015, Graz

S1 Area extension modulus estimation

The dependence of bilayer thickness on osmotic pressure P is accounted for via the area extension modulus K_A and given by the equation[1]

$$d_B(P) = d_B(0)\frac{K_A + P\,d(P)}{K_A + P\,d_B(0)}. \tag{S1}$$

We estimated K_A for our coexisting liquid phases based on published data for single lipids and binary lipid mixtures by Rawicz *et al.*[2,3] The Ld phase under investigation consists essentially of DOPC, with approximately 10 mol% cholesterol.[4] Interpolating linearly between the two published values for 0 and 50 mol% cholesterol in DOPC[2] yields $K_A(\text{Ld}) = (430 \pm 30)\,\text{mN}\,\text{m}^{-1}$.

In the coexisting Lo phase, the main constituent is the saturated lipid DSPC, which is accompanied by ca. 30 mol% cholesterol.[4] Unfortunately, published K_A values for saturated lipids are sparse. As a compromise, we interpolated linearly between pure DMPC (0 mol% cholesterol) and a 1:1 mixture of sphingomyelin/cholesterol,[2,3] yielding $K_A = (2100 \pm 500)\,\text{mN}\,\text{m}^{-1}$ for our Lo phase.

As pointed out in the section *X-ray measurements* of the main text, knowing the magnitude of K_A is more important than getting the precise number. That is because the biggest estimated change in bilayer thickness turned out to be just 0.3 Å. In principle, such a subtle difference in d_B would be resolvable with SAXS, but not with the additional scattering signal due to PEG.

S2 Finite size convergence

With open edges, one generally expects a 'surface' perturbation proportional to the relative size of the boundary to the interior, i.e. proportional to $1/N$ for our systems. As is

well known, periodic boundary conditions generally reduce this perturbation. They also speed up the convergence with system size, from $1/N$ to $1/N^2$ in a case well documented by Bonner and Fisher[5] (note their Fig. 1) and in the case of the one-dimensional Ising model the convergence is exponentially fast with periodic boundary condions. While another case with very slow convergence is known,[6] that one is due to very long range interactions not present in our membrane stacks. For periodic boundary conditions, the exact solution of a harmonic approximation to Eq. (3) suggests that d_W and Δ converge asymptotically like $y(N) \sim c_\infty - c_2/N^2$, i.e. convergence is expected to be faster than $1/N$ and, in agreement with the previous simulations,[7] our results are consistent with a dominant $1/N^2$ asymptotic convergence, allowing, of course, for higher order terms.

We perform simulations for several 'densities' $N \in \{N_{min}, \dots, N_{max}\}$ and fit them with the function $y(N) = c_\infty + \sum_{k=2}^{k_{max}} c_k/N^k$. Together with the originally proposed $k_{max} = 3$ and $N \in \{6, \dots, 32\}$, this method yields sufficiently precise continuum estimators c_∞, compared to the experimental uncertainties.[8] However, we found that varying the arbitrary parameters k_{max} and N_{min} influenced the final estimator stronger for some simulations (e.g. high pressures) than for others. To obtain more reliable uncertainties and perhaps even better continuum estimates, we perform now several extrapolations, with different values for k_{max} and N_{min}, but always using the highest possible N_{max}. By not changing N_{max}, we weight the most significant simulations (with the highest density) stronger. This procedure yields a list of results for $c_{\infty,l}$, which we average for the final estimator. Its uncertainty is then determined by the individual errors of $c_{\infty,l}$ (statistical uncertainty of observables due to finite simulation length) and their standard deviation (error due to finite simulation density). This procedure is closely related to the Jackknife technique.[9,10]

Comparisons between these improved Jackknife estimators and estimators obtained by the original method are given in Fig. S1. The relative difference in the estimators were less than 5% for all performed simulations, but most importantly, Jackknife produces a meaningful uncertainty.

S3 Efficient differentiation

A single simulation of a particular set of parameters $\vec{\Lambda} = (P, A, H, \lambda, K_c, \dots)$ contains more information in the generated time series, than the aforementioned observables which are determined by averaging. By reweighting the simulated histogram of density of states, it is possible to compute these quantities over a certain range of simulation parameters and thereby also derive their gradients.[11–13 14] This well recognized method was briefly mentioned for membrane MC simulations,[15] but has not been implemented for them previously.

We calculated the expectation value of an observable $f(u, \bar{a})$ for a different set of parameters $\vec{\Lambda}'$ from a simulation performed at $\vec{\Lambda}$ by

$$\langle f \rangle_{\vec{\Lambda}'} = \frac{\sum f_{\vec{\Lambda}'}(u, \bar{a}) \cdot \exp\left(-\delta G/kT\right)}{\sum \exp\left(-\delta G/kT\right)}, \tag{S2}$$

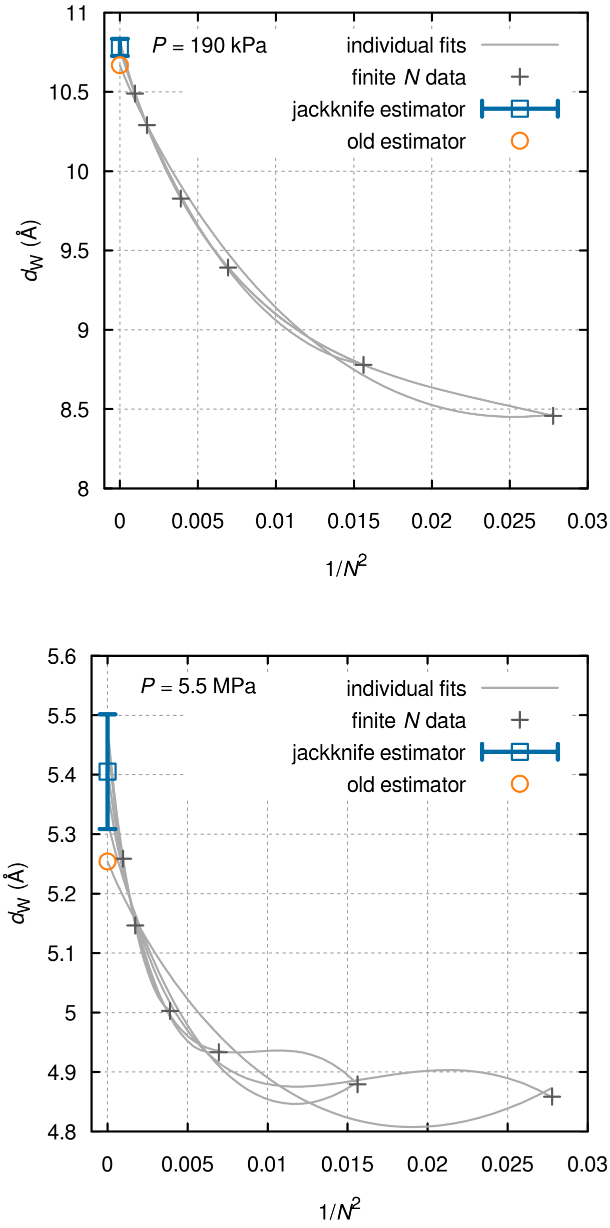

Figure S1: Finite size convergence of membrane spacing d_W vs membrane "density" N of Ld domains according to Tab. 1 at intermediate (top) and high osmotic pressures P (bottom). A variant of Jackknife allows us to obtain reasonable errors for the estimator. Statistical uncertainties for plotted finite N data are less than 10^{-2} Å.

where the sums extend over all realized configurations and δG is the change in the Gibbs energy of each state $(u, \bar{a})$ upon changing $\vec{\Lambda}$ to $\vec{\Lambda}'$. Most parameters could be separated from u and $\bar{a}$ in our case, yielding $\delta G(u, \bar{a}) = \delta\Lambda \cdot \xi(u, \bar{a})$. This allowed us to store only the time series of ξ instead of all realized states. The parameters P, A, H, and K_c were separable in this way, yielding

$$\frac{\delta G}{V} = \delta P\, \xi_P + \delta A\, \lambda\, \xi_A - \frac{\delta H}{12\pi}\, \xi_H + N^2 \frac{\delta K_c}{2}\, \xi_{Kc}, \tag{S3}$$

where $\xi_P = \bar{a}/d_W$, $\xi_A = \overline{\exp(-a/\lambda)}$, $\xi_H = \overline{1/a^2}$, and $\xi_{Kc} = \overline{q^4 |u_m(q_x, q_y)|^2}$. The local distance between membranes is denoted by $a = u_{m+1}(x, y) - u_m(x, y) + \bar{a}$, while the bars denote averages over (m, x, y) or (m, q_x, q_y). $V = L^2 M \bar{a}$ is the membrane stack's volume.

Separating λ from $(u, \bar{a})$ in δG turned out to be impossible, but we were able to calculate gradients of d_W and Δ with respect to λ efficiently. Because d_W and Δ did not depend explicitly on λ (i.e. $\partial f/\partial\lambda = 0$), differentiating Eq. (S2) yielded,

$$\left.\frac{\partial \langle f \rangle_{\lambda'}}{\partial \lambda'}\right|_{\lambda'=\lambda} = -\frac{AV}{kTN^2\Omega} \left(\sum f(u, \bar{a}) \xi_\lambda - \langle f \rangle_\lambda \sum \xi_\lambda \right), \tag{S4}$$

where sums extend over all realized states, Ω denotes the collection length and

$$\xi_\lambda = \overline{\left(1 + \frac{a}{\lambda}\right) \exp\left(-\frac{a}{\lambda}\right)}. \tag{S5}$$

Up to first order, $\langle f \rangle_{\lambda'}$ was then determined from $\langle f \rangle_{\lambda'} \approx \langle f \rangle_\lambda + (\lambda' - \lambda)\, \partial\langle f \rangle/\partial\lambda$.

Thus, for any observable $f \in \{d_W, \Delta\}$ and parameter $\Lambda \in \{P, A, H, K_c, \lambda\}$, we first determined $\langle f \rangle_{1,2}(N)$ for $\Lambda_{1,2} = \Lambda \pm \delta\Lambda$ as detailed above, extrapolated these expectation values for $N \to \infty$ according to section S2, and finally calculated the finite difference quotient $\partial\langle f \rangle/\partial\Lambda \approx (\langle f \rangle_1 - \langle f \rangle_2)/2\delta\Lambda$. Relative finite differences were set to $\delta\Lambda/\Lambda = 0.03$.

We checked this method against direct numerical differentiation for a couple of reasonable parameters. Errors were always sufficiently small (well below 50%) to lead the optimization routine towards a global minimum (see the section *Optimizing parameters against experimental data* of the main text).

S4 Results for a homogeneous control sample

We tested our analysis on already published SAXS data for homogeneous DMPC MLVs determined at 30 °C.[16] The Lifshitz calculation of the van der Waals forces yielded a value of $H = 4.11$ zJ for the published bilayer thickness of 44.0 Å. The obtained values describing the intersurface forces are given in Tab. S1, while Fig. S2 compares the simulations with the experimental data. Reassuringly, the simulations fit the experimental osmotic pressure data well. While the fit to Δ is excellent for high hydration, the fit becomes relatively poor for Δ as d_W becomes small, similarly to our L_o sample and likely for the same reason given in the main text.

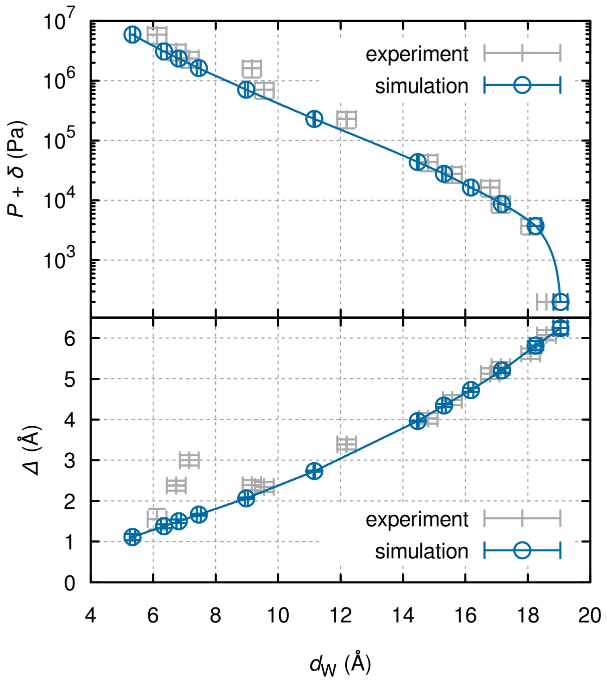

Figure S2: Osmotic pressure (top) and fluctuations (bottom) vs water-layer thickness for best fit of membrane MC simulation (cyan) against SAXS data (light gray) obtained from Ref. 16.[17] Solid lines were obtained by exponentially interpolating fluctuation contributions.

The interaction parameters obtained from the fit are shown in Tab. S1. Literature values for DMPC's bending modulus range from 50–130 zJ at 30 °C.[18] In light of this large variation, comparing only results of related methods is appropriate. Ref. 16 could not determine K_c and the modulus B separately and therefore considered several values of K_c; two of these are shown in Tab. S1. The values of A agree very well with ours. The larger values of λ would have been smaller if the true value of K_A had been known at that time. Two differences from the previous analyses are that here we calculated H and we used simulations; these cause the main differences reflected in the pairs of values for H and K_c in Tab. S1. Table S1 also shows results from another study,[19] that employed the same kind of simulations used here and differed by obtaining X-ray data from oriented stacks of DMPC bilayers, from which K_c was obtained directly. It also used the same P data, but failed to readjust the A and λ values to account for the corrected K_A. Nevertheless, agreement is reasonable.

Table S1: Optimal parameters found for describing the DMPC data published in Ref. 16.

	Current	1998a[16]	1998b[16]	2005[19]
H/zJ	4.11	7.13	4.91	6.1
K_c/zJ	57 ± 5	50	80	69
A/Pa	$10^{8.1 \pm 0.2}$	$10^{8.1}$	$10^{8.1}$	$10^{8.1}$
λ/Å	1.66 ± 0.15	1.91	1.97	1.91

For completeness, the functional dependence of the individual fundamental surface forces for DMPC is plotted in Fig. S3. The fluctuation force becomes the dominant repulsive force when d_W exceeds 9 Å, intermediate between the values of the Ld and Lo phases in Fig. 7, suggesting that the DMPC bilayer fluctuations are intermediate in this regard between the more fluid Ld phase and the more ordered Lo phase in the studied mixture. This is consistent with the Ld phase having a high concentration of the more disordered unsaturated lipids and the Lo phase having longer saturated chains with cholesterol.

S5 SAXS analysis

Comparisons between full q-range SAXS analyses and experimental data are shown in Fig. S4. Deviations between data and fits, especially for higher q ranges, are due to imperfect background subtraction, as explained in the section *X-ray measurements* in the main text.

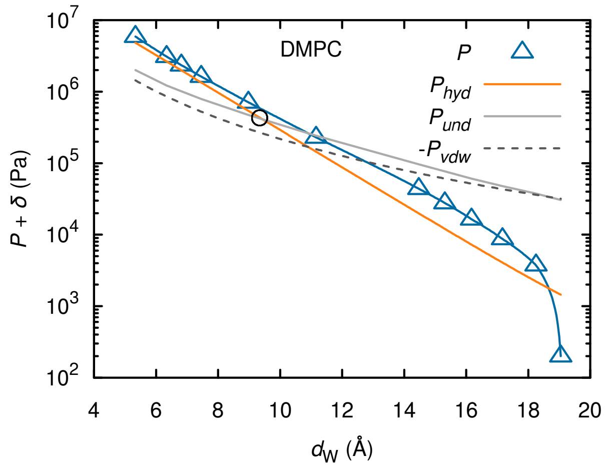

Figure S3: Partitioning of total pressure P into contributions from hydration P_{hyd}, van der Waals P_{vdw}, and undulations P_{und} for DMPC.[17] The large open black circle shows the value of the separation d_W at which hydration and undulation pressure are equal.

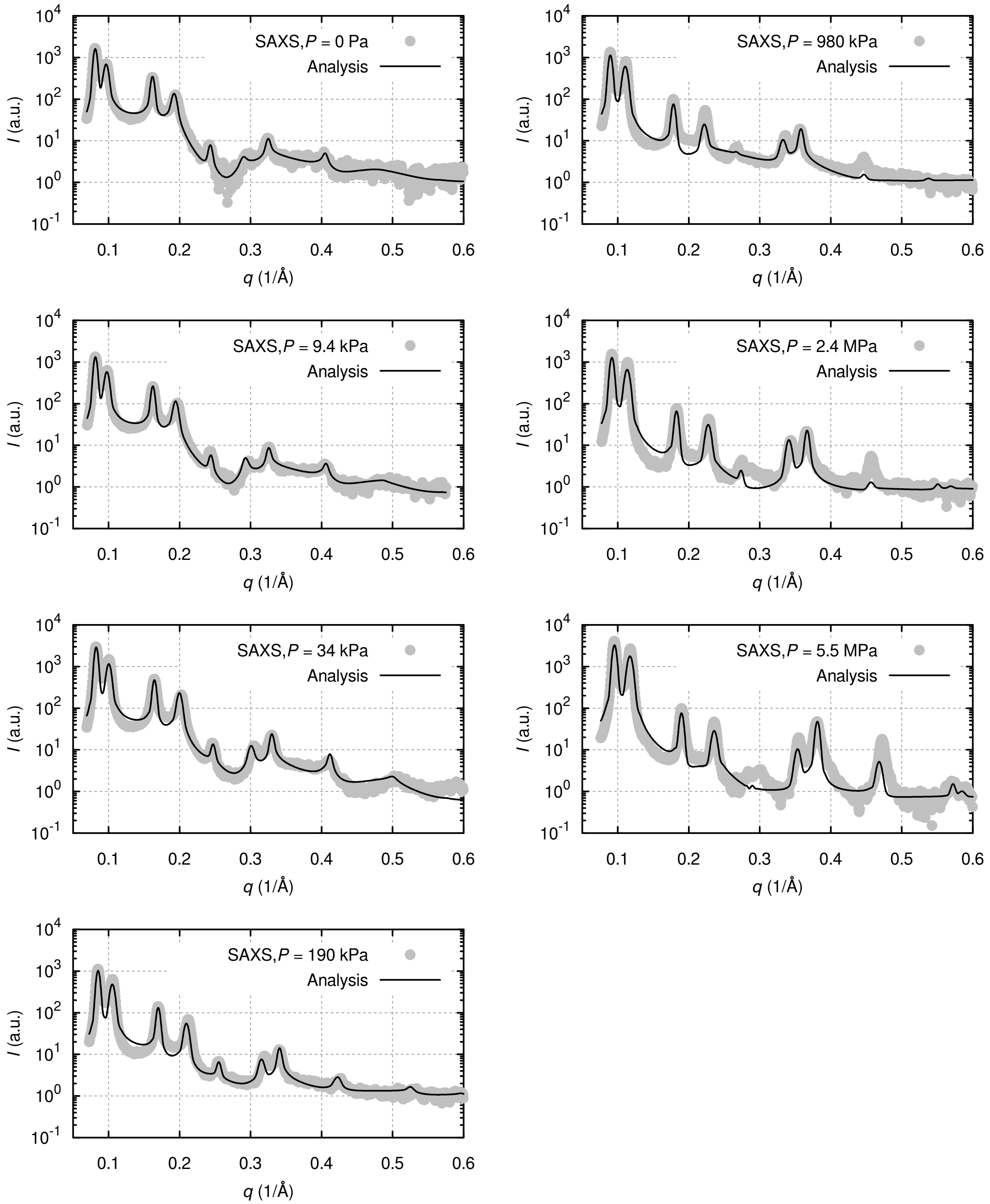

Figure S4: Calculated scattering intensities (solid lines) from full q-range analyses, compared to recorded SAXS data from coexisting phases (dots) of DOPC/DSPC/Chol (0.42:0.37:0.21), for all recorded osmotic pressures P.

S6 Fluctuations of the interbilayer water spacing

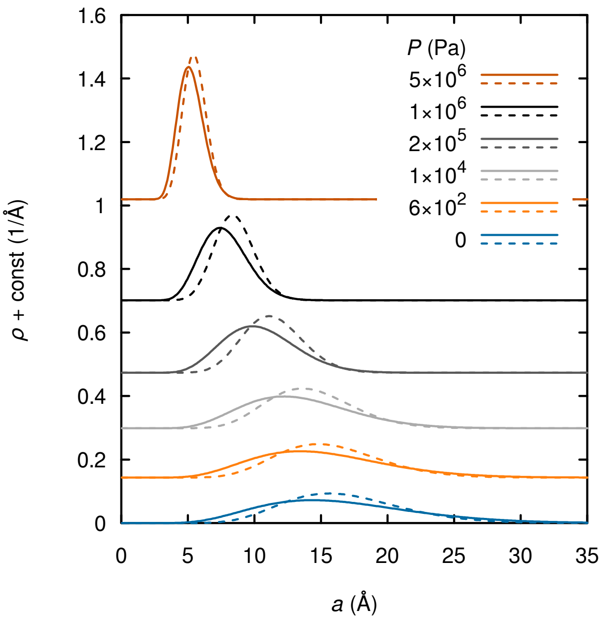

Figure S5: Probability density function ρ of the water spacing a at different external pressures P, for Ld (solid) and Lo (dashed) according to Tab. 1, obtained from $N = 32$ simulations.

References

[1] R. P. Rand and V. A. Parsegian. Hydration Forces Between Phospholipid-Bilayers. *Biochim. Biophys. Acta*, 988(3):351–376, December 1989.

[2] W. Rawicz, B.A. Smith, T.J. McIntosh, S.A. Simon, and E. Evans. Elasticity, Strength, and Water Permeability of Bilayers that Contain Raft Microdomain-Forming Lipids. *Biophys. J.*, 94(12):4725–4736, June 2008.

[3] W. Rawicz, K. C. Olbrich, T. McIntosh, D. Needham, and E. Evans. Effect of Chain Length and Unsaturation on Elasticity of Lipid Bilayers. *Biophys. J.*, 79(1):328–339, July 2000.

[4] Frederick A. Heberle, Jing Wu, Shih Lin Goh, Robin S. Petruzielo, and Gerald W. Feigenson. Comparison of Three Ternary Lipid Bilayer Mixtures: FRET and ESR Reveal Nanodomains. *Biophys. J.*, 99(10):3309–3318, November 2010.

[5] Jill C. Bonner and Michael E. Fisher. Linear Magnetic Chains with Anisotropic Coupling. *Phys. Rev.*, 135(3A):A640–A658, August 1964.

[6] J. F. Nagle and J. C. Bonner. Numerical studies of the Ising chain with long-range ferromagnetic interactions. *J. Phys. C: Solid State Phys.*, 3(2):352, February 1970.

[7] Nikolai Gouliaev and John F. Nagle. Simulations of Interacting Membranes in the Soft Confinement Regime. *Phys. Rev. Lett.*, 81(12):2610–2613, September 1998.

[8] Nikolai Gouliaev and John F. Nagle. Simulations of a single membrane between two walls using a Monte Carlo method. *Phys. Rev. E*, 58(1):881–888, July 1998.

[9] M. H. Quenouille. Notes on Bias in Estimation. *Biometrika*, 43(3/4):353–360, December 1956.

[10] John W. Tukey. Bias and confidence in not quite large samples (abstract). *Ann. Math. Statist.*, 29(2):614, June 1958.

[11] Z. W. Salsburg, J. D. Jacobson, W. Fickett, and W. W. Wood. Application of the Monte Carlo Method to the Lattice-Gas Model. I. Two-Dimensional Triangular Lattice. *J. Chem. Phys.*, 30(1):65–72, January 1959.

[12] Alan M. Ferrenberg and Robert H. Swendsen. New Monte Carlo technique for studying phase transitions. *Phys. Rev. Lett.*, 61(23):2635–2638, December 1988.

[13] Alan M. Ferrenberg and Robert H. Swendsen. Optimized Monte Carlo data analysis. *Phys. Rev. Lett.*, 63(12):1195–1198, September 1989.

[14] A common extension of this method would even allow to combine information from multiple simulations (WHAM)[20].

[15] Nikolai Gouliaev. *Monte-Carlo simulations of membrane systems.* PhD thesis, Carnegie Mellon University, Pittsburgh, Pennsylvania, USA, July 1998.

[16] Horia I. Petrache, Nikolai Gouliaev, Stephanie Tristram-Nagle, Ruitian Zhang, Robert M. Suter, and John F. Nagle. Interbilayer interactions from high-resolution x-ray scattering. *Phys. Rev. E*, 57(6):7014–7024, June 1998.

[17] The small offset $\delta = 200\,\mathrm{Pa}$ is necessary for plotting $P = 0$.

[18] John F. Nagle. Introductory Lecture: Basic quantities in model biomembranes. *Faraday Discuss.*, 161:11, 2013.

[19] Nanjun Chu, Norbert Kučerka, Yufeng Liu, Stephanie Tristram-Nagle, and John F. Nagle. Anomalous swelling of lipid bilayer stacks is caused by softening of the bending modulus. *Phys. Rev. E*, 71(4):041904, April 2005.

[20] Shankar Kumar, John M. Rosenberg, Djamal Bouzida, Robert H. Swendsen, and Peter A. Kollman. The weighted histogram analysis method for free-energy calculations on biomolecules. I. The method. *J. Comput. Chem.*, 13(8):1011–1021, 1992.
